# Evolution of substrate recognition sites (SRSs) in cytochromes P450 from Apiaceae exemplified by the CYP71AJ subfamily

**DOI:** 10.1186/s12862-015-0396-z

**Published:** 2015-06-26

**Authors:** Bjørn Dueholm, Célia Krieger, Damian Drew, Alexandre Olry, Tsunashi Kamo, Olivier Taboureau, Corinna Weitzel, Frédéric Bourgaud, Alain Hehn, Henrik Toft Simonsen

**Affiliations:** University of Copenhagen, Department of Plant and Environmental Science, Copenhagen Plant Science Centre, Thorvaldsensvej 40, 1871 Frederiksberg C, Denmark; University of South Australia, School of Pharmacy and Medical Sciences, Adelaide, South Australia; Technical University of Denmark, Centre for Biological and Sequence Analysis (CBS), Anker Engelunds Vej 1, 2800 Kgs. Lyngby, Denmark; UMR1121 Université de Lorraine, 2 Avenue de la Forêt de Haye, 54518 Vandoeuvre-les-Nancy, France; UMR1121 INRA, 2 Avenue de la Forêt de Haye, 54518 Vandoeuvre-les-Nancy, France; Molécules Thérapeutiques in silice (MTi), Inserm UMR-S 973 - Université Paris Diderot, Bat Lamarck A, 35 Rue Hélène Brion, 75205 Paris, France; National Institute for Agro-Environmental Sciences, 3-1-3 Kan-nondai, Tsukuba, 305-8604 Ibaraki Japan

**Keywords:** Cytochrome P450, Furanocoumarin, Hydroxycoumarin, Apiaceae, Apioideae, Gene duplication, Plant-insect coevolution, CYP71AJ subfamily, Substrate recognition sites (SRSs), *Pastinaca sativa*

## Abstract

**Background:**

Large proliferations of cytochrome P450 encoding genes resulting from gene duplications can be termed as ‘blooms’, providing genetic material for the genesis and evolution of biosynthetic pathways. Furanocoumarins are allelochemicals produced by many of the species in Apiaceaous plants belonging to the Apioideae subfamily of Apiaceae and have been described as being involved in the defence reaction against phytophageous insects.

**Results:**

A bloom in the cytochromes P450 CYP71AJ subfamily has been identified, showing at least 2 clades and 6 subclades within the CYP71AJ subfamily. Two of the subclades were functionally assigned to the biosynthesis of furanocoumarins. Six substrate recognition sites (SRS1-6) important for the enzymatic conversion were investigated in the described cytochromes P450 and display significant variability within the CYP71AJ subfamily. Homology models underline a significant modification of the accession to the iron atom, which might explain the difference of the substrate specificity between the cytochromes P450 restricted to furanocoumarins as substrates and the orphan CYP71AJ.

**Conclusion:**

Two subclades functionally assigned to the biosynthesis of furanocoumarins and four other subclades were identified and shown to be part of two distinct clades within the CYP71AJ subfamily. The subclades show significant variability within their substrate recognition sites between the clades, suggesting different biochemical functions and providing insights into the evolution of cytochrome P450 ‘blooms’ in response to environmental pressures.

**Electronic supplementary material:**

The online version of this article (doi:10.1186/s12862-015-0396-z) contains supplementary material, which is available to authorized users.

## Background

Many scientific articles report that coevolution between plant and their predators is a matter of arms race. The biosynthesis of specialized metabolites in plants produces a vast array of defensive allelochemicals to cope with herbivores and pathogens [1]. On their side, many insect herbivores have evolved the ability to metabolize these defence compounds, thus have adapted to overcome much of the toxicity [2, 3].

**Fig. 1:**
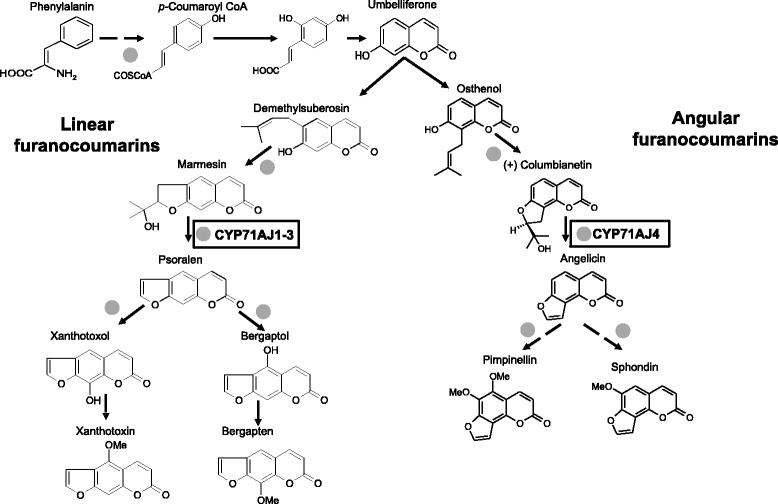
Simplified furanocoumarin biosynthesis pathway. Grey dots corresponds to demonstrate or putative cytochrome P450 dependent steps

Furanocoumarins are molecules often used to illustrate this coevolution. Two kinds of furanocoumarins are described, which differ by the position of a furan group grafted on a coumarin core molecule either at position C6-C7 for linear molecules or C7-C8 for the angular one (Fig. 1). Due to their chemical structure, the linear molecules are highly toxic to a broad spectrum of predators. Although angular molecules are less toxic, the resistance spectrum is expanded when the plants simultaneously produce both linear and angular structures. These kinds of molecules have been reported to exist predominantly in four plant families: Rutaceae, Moraceae, Fabaceae and Apiaceae (for review see [4]). The distribution of linear and angular furanocoumarins differs between each of these four plant families, highlighting a difference in their evolutionary path. In particular, only linear furanocoumarins have been reported in Rutaceae and Moraceae, while both linear and angular furanocoumarins have been described in Fabaceae. It is important to note that no plant has so far been identified that produces only angular molecules, which has led some authors to conclude that linear furanocoumarins appeared earlier during evolution and that the rise of the angular molecules was related to a strengthening of the defensive capacity of plants with respect to the attack by phytopathogenic insects [5]. Apiaceae, the fourth family to produce furanocoumarins, is an interesting case study since they comprise the 3 possible evolutionary stages related to the occurrence of furanocoumarins in higher plants: (i) some species of Apiaceae are unable to synthesize these molecules, (ii) others are able to synthesize only linear furanocoumarins, (iii) and others contain both linear and angular molecules. Within Apiaceae the subfamily Apioideae is the largest of the four subfamilies described in Apiaceae consisting of more than 2900 species and more than 400 genera [6, 7], even though many of these genera are not well resolved [8]. The other subfamilies are Azorelloideae, Mackinlayoideae, and Saniculoideae, and more than 180 species in Apiaceae have not been classified in sub-families but have been classified in tribes only [6, 9–11].

The understanding of the furanocoumarin biosynthesis is not yet fully resolved [4]. Investigations carried out in the late eighties by Hamerski and colleagues on *Ammi majus* cell cultures using radioactive precursors demonstrated that many steps in the biosynthesis of these molecules are catalyzed by enzymes of the cytochrome P450 family [12]. Recently a cytochrome P450 subfamily has been described with members existing in parsnip (*Pastinaca sativa*), celery (*Apium graveolens* var. *dulce*) and *Ammi majus,* which has been shown to be involved in the synthesis of linear and angular furanocoumarins. CYP71AJ1-3 were identified as psoralen synthases and CYP71AJ4 has been characterized as an angelicin synthase [13, 14] (Fig. 1). Both enzymes catalyze the same kind of reaction, cleaving the C-C bond at the C-3’ position forming the furanocoumarin skeleton and acetone in equimolar amounts [15, 16].

**Fig. 2:**
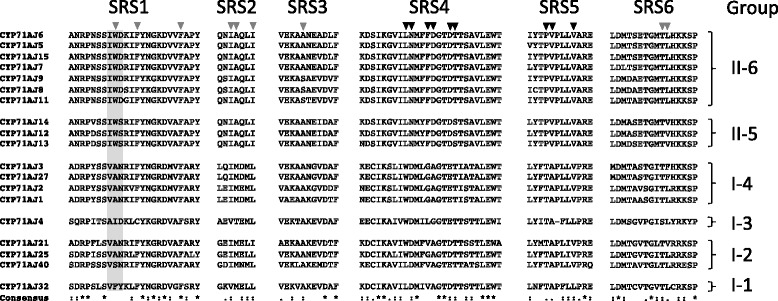
SRS alignment of 19 cytochrome P450 belonging to the CYP71AJ subfamily. SRS means Substrate Recognition Site. The three-amino-acid motif is highlighted with a grey box. Residues likely to constitute the active site are indicated with triangles, and those in black are considered more reliable for their position in the homology models than those in grey

Despite low sequence similarity, the overall structure of cytochromes P450 is generally conserved and six substrate recognition sites (SRS1-6) have been identified to be necessary in the active site [17]. Thus, the SRS1-6 regions are very important for the binding and the subsequent enzymatic conversion of the substrate (Fig. 2) [18–20]. Other characteristic regions of plant and animal cytochrome P450s are the proline rich membrane hinge, the sequence surrounding the cysteine that is the axial ligand to the heme, the I-helix (i.e. SRS4) involved in oxygen binding, and the E-R-R triad using the Glu and Arg of the K-helix consensus (**E**xx**R**) and the Arg in the “PE**R**F” consensus. The E-R-R triad is generally thought to be involved in locking the heme pocket into position and to ensure stabilization of the conserved core structure [17].

For several molecules, including 2,4-dihydroxy-7-methoxy-1,4-benzoxazin-3-one (DIMBOA), it has been reported that allelic variants of a single cytochrome P450 subfamily are involved in each step of the biosynthetic pathway [21]. Concerning the furanocoumarin pathway, already two enzymes belonging to the same sub-family (CYP71AJ) catalyze the synthesis of two different molecules. It is therefore conceivable that other enzymes belonging to the same subfamily are able to catalyze some other steps. To confirm or reject this assumption, we explored the evolution of this cytochrome P450 subfamily through the study of 38 CYP71AJ coding sequences. In addition to the already described CYP71AJ1-4, 34 new sequences were identified through experimental approaches or data mining of transcriptomic libraries in various Apiaceae. The identification of genes encoding CYP71AJ in plants that do not produce furanocoumarins provide evidence that this enzyme family is likely to play a broader role in the metabolism of these plants and probably result from a complex evolution process. This is confirmed by the absence of furanocoumarin (or derivative) metabolisation of some corresponding proteins and by an *in silico* analysis of the SRS1-6 regions which highlights residues important for accepting linear or angular dehydrofuranocoumarins or other substrates.

## Results and discussion

### CYP71AJs isolated from *Pastinaca sativa*

*Pastinaca sativa* is an Apiaceous plant able to synthesize both linear and angular furanocoumarins. In order to decipher the biosynthesis pathway of these molecules at the molecular level, we searched for CYP71AJ orthologous genes in an RNA-seq database generated from mRNA extracted from parsnip eight week old plantlets. In addition to CYP71AJ3 and -AJ4, which were respectively assigned to psoralen and angelicin synthases [13, 14], we identified a new gene, *cyp71aj13* encoding an enzyme sharing 76 % and 75 % identity with CYP71AJ3 and -AJ4, respectively. This new coding sequence was cloned into the pYeDP60 expression vector and the corresponding protein expressed in yeast in order to functionally characterize the enzyme. To examine that the protein was properly expressed, we added a tag of 6xHis to the C-terminus and performed a western blot prior to any enzymatic functional investigation. A metabolic screening was performed using 30 different furanocoumarins (Additional file 1) including 18 linear molecules and 12 angular molecules. Since none of them was metabolized, we extended the screening to 23 additional coumarins and 3 hydroxycinnamic acids (Additional file 1) with no more success. These first experimental results suggest that not all the enzymes belonging to this CYP71AJ cytochrome P450 subfamily share the same substrate specificity. To investigate this point, we compared the peptidic sequence of this enzyme and the sequence of enzymes whose function has already been established ie CYP71AJ3 and CYP71AJ4. Although, the overall sequence identity is close to 75 % between CYP71AJ13 and psoralen synthase/angelicin synthase, it decreases significantly, when the comparison is focused on the substrate recognition sites (SRSs) conserved in cytochrome P450 [18]. This analysis shows that CYP71AJ13 is closer to CYP71AJ3 than CYP71AJ4 (Table 1 and Fig. 2) especially for SRS1, SRS5, and SRS6, which are well conserved between CYP71AJ13 and CYP71AJ3. However, 3 triple mutation lead to important modifications in SRS1, SRS6, and SRS4 and may change the substrate specificity of these enzymes. In addition, Larbat and collaborators assumed that SRS2 and SRS3 form an access channel for the substrate to the active site [4, 14]. Whereas SRS3 doesn’t display significant changes between the three enzymes, a detailed analysis of SRS2 highlights differences of almost all the amino acid residues. For example, a modification of Leu204/Ala212 in CYP71AJ3/4 (which is a Gln in CYP71AJ13), Met207/Thr215 in CYP71AJ3/4 (which is an Ala in CYP71AJ13) or Met217 in CYP71AJ3-4 (which is Leu in CYP71AJ13) will affect the access to the active site of the enzyme. Finally, the appearance of a proline (Pro370) in CYP71AJ13 instead of a threonine (Thr361/Thr368) in CYP71AJ3/4 might involve a structural modification in SRS5 and probably also impact the substrate specificity of this enzyme.

**Table 1:** Sequence comparison of SRS identified in CYP71AJ13, psoralen synthase (CYP71AJ3) and angelicin synthase (CYP71AJ4). Results are presented as percentage identity

	SRS 1	SRS 2	SRS 3	SRS 4	SRS 5	SRS 6
CYP71AJ3	62	30	67	38	58	64
CYP71AJ4	43	10	67	38	33	29

### The bloom in the CYP71AJ subfamily

The lack of metabolisation of a wide range of coumarins or derivatives and the presence of many differences within SRS of CYP71AJ13 in comparison to CYP71AJ3/4 suggest a functional diversification within this P450 subfamily. As furanocoumarins have appeared lately in certain plants to defend against attacks by predators, we assume that genes involved in the synthesis of these molecules are deriving from older genes displaying other functions. Among the Apiaceae, it has been reported that some genera could produce furanocoumarins while others could not. Therefore we investigated the occurrence of orthologous genes in different Apiaceae transcriptomic libraries available in databases by using BLAST searches. This investigation, in addition to an experimental PCR-based fishing approach using primers targeted toward conserved sequences, led us to identify 34 new sequences encoding CYP71AJ enzymes in 19 different apiaceous plant species. Among the 34 coding sequences identified this way, 15 were considered as full-length and were used in addition to the already described CYP71AJ1-4 for subsequent *in silico* investigations (Table 2 for full-length and Additional file 2). In addition to the plants listed in Table 2, mRNAs were also extracted from other Apiaceae species, namely *Crithmum maritimum* L. (Pyramidoptereae Boiss. clade), *Pimpinella cretica* (Pimpinelleae Spreng. clade), *Oenanthe divaricate* (Oenantheae Dumort. clade), and *Eryngium campestre* as well as *Sanicula europaea* (both in subfamily Saniculoideae), but in these species no CYP71AJ sequences were identified using the PCR approach with the primers described here (Additional file 3).

**Table 2:** Full-length CYP71AJ members included in the dN/dS selection tests

Name	Species (clade)	Functionality/group
CYP71AJ1	*Ammi majus* L. (Apieae)^a^	Psoralen synthase/I-4
CYP71AJ2	*Apium graveolens* L. (Apieae)^a^	Psoralen synthase/I-4
CYP71AJ3	*Pastinaca sativa* L. (Tordylieae, Tordyliinae)^a^ ^b^	Psoralen synthase/I-4
CYP71AJ4	*Pastinaca sativa* L. (Tordylieae, Tordyliinae)^a^ ^b^	Angelicin synthase/I-3
CYP71AJ5	*Thapsia garganica* L. (Daucinae, Scandiceae)	Unknown/II-6
CYP71AJ6	*Thapsia laciniata* Rouy, (Daucinae, Scandiceae)^b^	Unknown/II-6
CYP71AJ7	*Daucus carota* ssp sativus (Daucinae, Scandiceae)	Unknown/II-6
CYP71AJ8	*Ammi majus* L. (Apieae)^a^	Unknown/II-6
CYP71AJ9	*Petroselinum crispum* Mill (Apieae)^a^	Unknown/II-6
CYP71AJ11	*Heracleum mantegazzianum* L. (Tordylieae, Tordyliinae)^a^	Unknown/II-6
CYP71AJ12	*Thapsia laciniata* L. (Daucinae, Scandiceae)^b^	Unknown/II-5
CYP71AJ13	*Pastinaca sativa* L. (Tordylieae, Tordyliinae)^a^	Unknown/II-5
CYP71AJ14	*Thapsia garganica* L. (Daucinae, Scandiceae)	Unknown/II-5
CYP71AJ15	*Laserpitium siler* L. (Daucinae, Scandiceae)	Unknown/II-6
CYP71AJ21	*Daucus carota* ssp sativus (Daucinae, Scandiceae)	Unknown /I-2
CYP71AJ25	*Thapsia garganica* L. (Daucinae, Scandiceae)	Unknown /I-2
CYP71AJ27	*Heracleum lanatum* Bertram. (Tordylieae, Tordyliinae)^a^	Putative psoralen synthase/I-4
CYP71AJ32	*Bupleurum chinense* L. (Bupleureae)^b^	Unknown/I-1
CYP71AJ36	*Apium graveolens* L.	Unknown/I-2

^a^Members of the apioid superclade. ^b^Used for the homology modeling

**Fig. 3:**
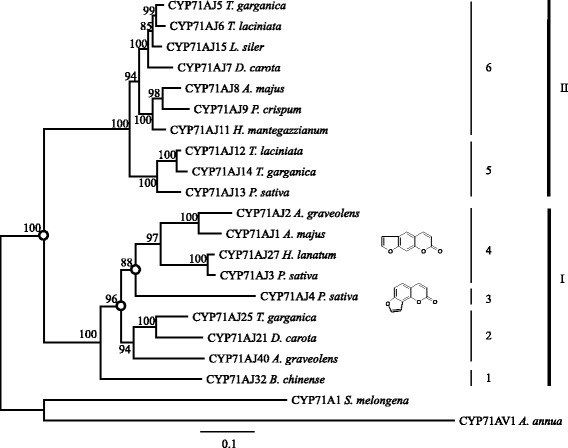
Phylogeny tree of 19 full length CYP71AJ. The tree is based on a MUSCLE alignment followed by personal fitting, then a tree building using LG within PhyML. All is performed in Geneious® 6.1.8. Reconstructed ancestral genes were analysed for notes indicated with dots. Alignment can be found in Additional file 4

Phylogenetic analyses of the 19 full-length CYP71AJs revealed that these enzymes are grouped in at least 2 large clades (I and II) including 6 subclades (Fig. 3). Eight enzymes are grouped on a first cluster, which is branched into two parts. The first contains enzymes whose involvement in the synthesis of furanocoumarins was already demonstrated (I-4 and I-3), and the second containing enzymes of unknown functions (I-2). This latter group includes a gene (*cyp71aj25*) isolated from *Thapsia garganica* which has been reported not to produce furanocoumarins, giving therefore arguments in favour of a functional diversification of CYP71AJs. The presence, in the same subclade, of genes isolated from *D. carota* and *A. graveolens* which are able to produce linear furanocoumarins, is consistent with the assumption that this enzyme subfamily has evolved from ancestral enzymes probably displaying different substrate specificity. Indeed, in this first clade, *A. graveolens* harbours two divergent coding sequences: CYP71AJ2 which was described as a psoralen synthase (belongs to I-4) and CYP71AJ36 (I-2) for which the function is still unknown.

A second clearly different clade could be identified (Clade II) with two different but sequential similar sub-clades. Similarly to the clade I, plants producing CYP71AJs in this second clade have been described as being either producing furanocoumarins or not. The previously tested CYP71AJ13 which was identified in *P. sativa*, a plant producing furanocoumarins, belongs to sub-clade II-5. Here we completed our study by carrying out the heterologous expression of two additional proteins, CYP71AJ5 and CYP71AJ6, identified in two Thapsia species which are reported not to produce furanocoumarins. A functional screening (using the same molecules used for CYP71AJ13 and described in the Additional file 1) performed on these proteins belonging to the group II-6 was unsuccessful thus reinforcing the hypothesis of a function not related to the synthesis of molecules close to coumarins.

### Evolution in the SRS regions of CYP71AJs

#### Substrate recognition sites in CYP71AJs

**Fig. 4:**
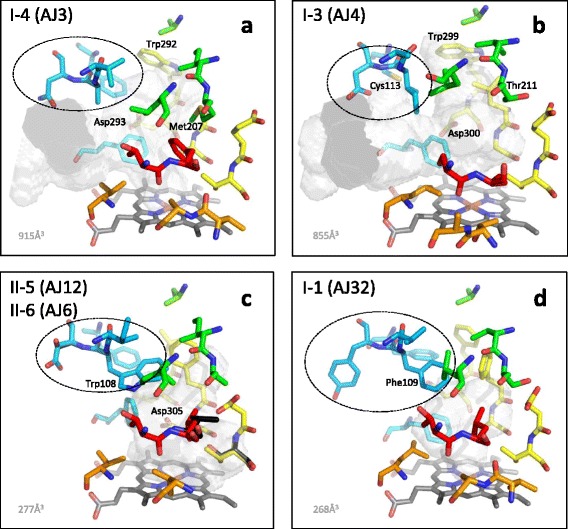
Potential active site configurations for five CYP71AJ groups. The homology models were constructed in CPHmodels-3.2 and VOIDOO was used for cavity calculations. SRS1: blue; SRS2 + 3: green; SRS4: yellow; SRS5: orange; SRS6: red; and heme-group: grey. The three-amino-acid motives in SRS1 that are likely important for substrate acceptance are indicated with dashed ovals. Residue differences between groups II-5 and II-6 are marked with black. Other residue likely to be important for the active sites are discussed in the text. **a**) CYP71AJ3, **b**) CYP71AJ4, **c**) CYP71AJ6 and CYP71AJ12, **d**) CYP71AJ32

Despite great variation between the enzymes in the cytochrome P450 superfamily, six substrate recognition sites (SRS1-6) have been identified constituting essential residues in the active site [18]. To investigate the potential relation between primary sequence and structure we generated homology models. The different CYP71AJ clades have distinct patterns in these six SRS regions. The SRS1 regions of the groups I-1 (VFY), I-3 (AID), II-5 (IWS), and II-6 (IWD) exhibit a “three-amino-acid” motif specific for each of these sub-clades, whereas group I-2 and I-4 share this motif (VAN) (boxed in Fig. 2**,** full sequence list in Additional file 2). These motif are likely be important for the substrate acceptance, and in the homology models (Fig. 4) the second residue in the motif is predicted to be in close proximity to the substrate after the final substrate orientation. The SRS1 motifs for groups II-5 and II-6 have larger residues than the residues of proteins functionally assigned to groups I-3 and I-4, which encode angelicin synthase and psoralen synthase, respectively. For all the groups, a proline localized beside amino acids harbouring large side chains (tyrosine and tryptophan) could play an important role in shaping the SRS1 region, particularly since proline residues structurally interrupt alpha helices [22]. Hydrophobic residues in the SRS1 region have been found to be conserved in cytochrome P450s described in insect herbivores feeding on furanocoumarin producing plants, e.g. in *Papilio* spp. feeding on apiaceous plants [23]. A similar pattern could be present in the apiaceous cytochromes P450 involved in coumarin biosynthesis.

SRS2 and SRS3 may be of lesser importance for the final structure of the substrate, but, as mentioned previously, could be important for substrate acceptance. The SRS4 (i.e. the I-helix) has many residues important for influencing the orientation of the substrate within the active site and for the transfer of the oxygen during product formation *via* a negatively charged residue (Asp/Glu) together with a polar uncharged side-chain (Ser/Thr) residue. Residues very likely to face inwards in the active site are indicated in Fig. 2.

The SRS5 region for group II-5 and II-6 are nearly identical, but the Pro-Val in these groups would make the active sites less polar than in the case of I-3 and I-4 where the equivalent residues are Thr-Ala. In group I-1 the SRS5 region is very similar to that of I-4 (psoralen synthases). The SRS6 region across the different groups also bears a high degree of similarity and the residues predicted to constitute the active site are not markedly different.

Some plant cytochrome P450 enzymes may be under co-evolutionary selection pressure with certain codon sites under positive selection, which especially would be interesting for codon sites within the SRS regions. Thus dN/dS selection analyses were performed with two different methods (REL [24, 25] and MEME [26]) to test for codon sites under positive but also negative (i.e. stabilizing) selection. The MEME method indicated that 20 sites are potentially under positive selection and three of the codon sites suggested by the REL method were congruent with these. These congruent amino acids were neither found to be in the SRS regions nor close to the interface involved in the interaction with a NADPH cytochrome P450 Reductase (CPR). They are thus likely false positives. For the remaining 17 codon sites found with the REL method, one was found within SRS2 (codon site 213, p-value 0.010), one within SRS3 (codon site 254, p-value 0.036), and one in SRS6 (codon site 488, p-value 0.018). These may not be residues critical for the substrate uptake and conversion; however, they might be an indication that these codon sites are under less stringent selection and mutations in these codons will have minor or no implication for the tertiary conformation of the CYP71AJ enzymes. Of the 31 codon sites suggested by the REL method to be under negative selection, two were found within SRS1 (codon sites 105 and 118, coding for Arg and Gly, respectively), one in SRS4 (codon site 302, coding for Ile), and one in SRS5 (codon site 378, coding for Pro). The tyrosine and tryptophan residues in SRS1 were not selected by the two methods.

To further investigate sites that might be under selection in the SRS regions, but additionally including the physiochemical properties of the residues, we performed two PRIME (PRoperty Informed Models of Evolution) analyses using five Conant-Stadler [27] properties (chemical composition, polarity, volume, iso-electric point, and hydropathy) and the five Atchley *et al.* [28] properties (polarity, secondary structure, volume, heat capacity, and iso-electric point). Both models found the residue coded at codon site 111 in SRS1 to have significant properties. Interestingly, this residue is the second residue in the three-amino-acid motif mentioned earlier (boxed in Fig. 2). The two models selected this residue on different criteria; the Conant-Stadler model based changing properties on chemical composition and conserved properties on iso-electric point, whereas the significance in the Atchley *et al.* properties model for changing properties was based on volume and conserved properties based on heat capacity [28]. The changing properties thus appear related to volume and chemical composition. The Conant-Stadler method found codon site 310 in SRS4 to be important based on changing properties in hydropathy, i.e. the hydrophilic and hydrophobic properties of the residue. This codon site does not code for one of the six residues suggested to be directly involved in the constitution of the active site, and the corresponding residue for this codon site in all 19 sequences are hydrophobic, thus not fully supporting the prediction. The Atchley *et al.* model found four putatively important residues in the SRS regions, two in both SRS2 (codon sites 213 and 216) and SRS6 (codon sites 489 and 496). The codon site 213 was an significant residue (p-value 0.003) based on changing properties in secondary structure, along with codon site 216 (p-value 0.042) based on conserved properties in polarity and iso-electric point. The latter, predicted by the homology models to constitute the active site, appears to differ at this point for group I-3 (Fig. 4b) and group I-4 (Fig. 4a) in that angelicin synthase (AJ4) has the polar residue Thr211 corresponding to the hydrophobic residue Met207 in psoralen synthase (AJ3). Both codon sites selected in SRS6 were based on conserved properties, and not predicted to constitute the active site (Additional file 5).

### Structural analysis

A full-length CYP71AJ (CYP71AJ32) and a fragment (CYP71AJ31) were identified from the Chinese species *Bupleurum chinense* using transcriptomic data. Fragments were also identified in *B. sacilifolium* (CYP71AJ33, PCR approach) and *B. scorzonerifolium* (CYP71AJ37, transcriptomic data). A phylogenetic analysis restricted to the partial protein sequences suggested that these four sequences group together in group I-1 (see Additional file 4 for the partial trees). When comparing I-1 to the other CYP71AJ groups many residues are conserved in the SRS1 region emphasizing the possible high structural importance of this site for the conformation of the substrate. The “three-amino-acid” motifs of the *Bupleurum* CYP71AJs are quite distinct from the other CYP71AJ members with VFY being present in CYP71AJ32 as opposed to VAN for the group I-2 and I-4, AID for the group I-3, IWS for the group II-5 and IWD for the group II-6. The active site volume of the CYP71AJ32 homology model was calculated to be 268 Å^3^ (Fig. 4d).

Homology modelling and subsequent docking has previously been carried out on psoralen synthase (group I-4) from *Ammi majus* (CYP71AJ1) to predict the binding mode of the (+)-marmesin substrate and the (+)-columbianetin, which acts as a competitive inhibitor [13]. The template structure for this model was CYP2C8 [PDB:1PQ2]. It was predicted that the (+)-marmesin substrate in the CYP71AJ1 homology model had its dehydrofuran-ring proximal to residues Ala297 and Thr301 in SRS4, Thr361 in SRS5, and Thr479 in SRS6. It was further predicted that Met120 and Val121 in SRS1 and Ala362, Leu365, Val366, and Pro367 in SRS5 enclosed the coumarin ring and positioned the C-3’ carbon atom of (+)-marmesin 3.78 Å from the iron-oxo center of the heme-group. Whereas, the distance to the C-3’ of (+)-columbianetin in a similar conformation was 6.27 Å and thus unlikely to undergo any reaction [13]. According to Larbat and collaborators [13], site-directed mutagenesis of Met120 to a Val residue in SRS1 of CYP71AJ1 didn’t change the enzymatic activity and therefore diminishes the putative importance of this residue. This is confirmed by our homology model of CYP71AJ3 in which Met120 faces outwards and seems to play a minor role in forming the active site in this psoralen synthase.

Interestingly for SRS1 residue Cys113 is only found in the angelicin synthase (group I-3, Fig. 4b), whereas other CYP71AJ groups have a Phe in this position. In the CYP71AJ4 homology model Phe121 together with Ile109 of the AID motif, appear to be the most important residues in SRS1. The Phe, however, is also present in the other CYP71AJ groups. Residues Met207 and Leu210 in SRS2 of the psoralen synthase seal the proximal part of the active site cavity from the heme-group, whereas the Met207 residue corresponds to Thr211 in the angelicin synthase, as mentioned earlier. This polar Thr residue in the angelicin synthase could interact with a hydroxyl-group in (+)-columbianetin. In SRS4 the residue-pair Trp-Asp in the N-terminal of the I-helix exists in both psoralen and angelicin synthases and might be involved in locking the coumarin core in place, with the Asp residue potentially forming hydrogen bonds. For the psoralen synthase in SRS4, the residue-pair Gly296-Ala297 constitute the cavity closest to the iron-oxo-heme group with the corresponding Leu303-Gly304 in the angelicin synthase. Residues Thr304 and Thr311 in SRS4 of CYP71AJ3 (group I-4) and -AJ4 (group I-3), respectively, could also play a role of hydrogen-bond formations to hydroxyl-groups of the dehydrofuranocoumarin substrates.

A conspicuous feature of group I-3 encoding the angelicin synthases is the missing Pro in the SRS5, which is present in all of the other CYP71AJ groups. Interestingly, this residue was predicted by the REL method to be under negative selection. This difference has the effect of reducing the distance of Thr368 from the iron-oxo-heme center in angelicin synthase compared to the corresponding Thr361 in psoralen synthase and possibly pushes Leu372 in angelicin synthase further into the active site compared to the corresponding Val366 in psoralen synthase. This is particularly important because changes in the SRS5 have been found to alter the substrate specificity for other plant P450 enzymes [29, 30].

Thr479 in SRS6 seems to be important for constituting the active site in CYP71AJ3 as it did in the CYP71AJ1 homology model constructed by Larbat and collaborators [13]. Ser485 takes the same spatial position in the homology model of CYP71AJ4 and is likely to play a similar role to that of the Thr in psoralen synthase, possibly also interacting with the isopropyloxy-group of the dehydrofuranocoumarin substrate. The whole active volume calculations for both models include a potential channel for the substrate uptake, and are thus estimated higher than the active sites alone. The size differences between CYP71AJ3 and -AJ4 are not considered markedly different though. The largest differences between these two functionally characterized CYP71AJ members are thus found in the “three-amino-acid” motif in SRS1, the central residue in SRS2, the second residue pair in SRS4, and in the deletion of Pro in SRS5 in angelicin synthase. It could be speculated that these changes alone could shift the functionality from one enzyme to the other.

Group I-2 members from the Scandiceae tribe, i.e. CYP71AJ21 from *Daucus carota* and CYP71AJ25 from *Thapsia garganica*, have their “three-amino-acid” motif in common with the psoralen synthases of group I-4 (i.e. motif-sequence VAN). Many residues likely to constitute the active site are also in common for the other SRS regions between these two groups. It could therefore be conceivable that CYP71AJ21 and –AJ25 are putative psoralen synthases. The third member of this group, i.e. CYP71AJ36 from *A. graveolens*, clearly has a strong homology with the characterized psoralen synthase (CYP71AJ2) from the same species; however, the “three-amino acid” motif differs on the second residue, which is predicted by PRIME and the homology models to be significant to the substrate uptake and binding.

The relatively high sequence similarity (and hence probable structural similarity) between the two groups makes it very likely that they metabolize the same or similar substrates. The active sites in the homology models of these groups were found to be 277 Å^3^ (Fig. 4c), and appear being smaller than the cavities of the psoralen synthase (Group I-4, 915 Å^3^, Fig. 4a) and angelicin synthase (Group I-3, 855 Å^3^, Fig. 4b). This probably results from larger hydrophobic residues such as Trp108 in SRS1, and Phe304 in SRS4, taking up considerable space of the active site cavities (Fig. 4c). Asp109 in CYP71AJ6 corresponds to Ser110 in CYP71AJ12 for the three-amino-acid motif in SRS1. In the SRS4 region Thr309 in CYP71AJ6 corresponds to Ser310 in -AJ12 for the residue-pair involved in the electron transfer, and Leu488 in CYP71AJ6 corresponds to Val489 in CYP71AJ12 located in SRS6. These are all minor differences but could be of importance since the substrates for the two novel groups seems to be different from the psoralen and angelicin synthases. For instance, the Val489 in SRS6 of the homology models is predicted to be just next to the Thr309 in SRS4. Residues Asp305 in SRS4 is very likely to play a crucial role in the site with a carboxyl acid group in close proximity (approx. 5-6 Å) to the iron-centre of the heme-group potentially being an active part in the product formation.

### CYP71AJ occurrence within the Apiaceae subfamily Apioideae

New putative members of both the psoralen and angelicin synthases have been identified (Additional file 4). Putative psoralen synthase sequences have been identified in *Seseli montanum* (CYP71AJ19), *Angelica archangelica* ssp *archangelica* (CYP71AJ20), *D. carota* ssp *sativus* (CYP71AJ21), *Ferula communis* ssp *glaucus* (CYP71AJ24), *Thapsia garganica* (CYP71AJ25), and in *Heracleum lanatum* (CYP71AJ27) and new putative angelicin synthases sequences, in addition to the one already functionally characterized from *Pastinaca sativa* (CYP71AJ4), have been identified in *Heracleum mantegazzianum* (CYP71AJ22), *A. archangelica* ssp *archangelica* (CYP71AJ23), and *H. lanatum* (CYP71AJ28). As expected, all these plants have been reported to produce furanocoumarins. In addition to these two sub-clades, we highlighted at least two novel subclades within the CYP71AJ subfamily in the Apioideae subfamily.

In some of the species investigated, multiple CYP71AJ paralogous genes have been identified. These include *P. sativa,* where in addition to psoralen synthase (CYP71AJ3) and angelicin synthase (CYP71AJ4), two new enzymes have been found; namely CYP71AJ10 (in group II-6, see Additional file 4) and CYP71AJ13 (in group II-5). The full-length sequence of CYP71AJ10, however, has not yet been obtained. Five CYP71AJ members were also identified in the transcriptome of *H. lanatum*: a group I-2 member (CYP71AJ26) putative psoralen synthase (CYP71AJ27), putative angelicin synthase (CYP71AJ28), a group II-6 member (CYP71AJ29), and a unique CYP71AJ sequence (CYP71AJ30) that seems to fall into group I-2 (see Additional file 4). Genera *Pastinaca* and *Heracleum* (both in tribe Tordyliinae) synthesize linear and angular furanocoumarins in high amounts. Genus *Angelica* (in tribe Selineae) synthesize linear furanocoumarins and angular dihydrofuranocoumarins predominantly with a few exceptions such as *A. angelica* ssp *archangelica*, which is also able to synthesize angular furanocoumarins [31]. Interestingly, the sequence comparison highlights that the angelicin synthase (CYP71AJ4 ortholog) found in this *Angelica* species is close to the angelicin synthases of *Pastinaca* (CYP71AJ4) and *Heracleum* (CYP71AJ4 orthologous, and CYP71AJ28). Given the phylogenetic distance between these genera, it is likely that the angelicin synthase gene could have occurred from a duplication/mutation of the psoralen synthase gene.

In *D. carota* ssp *sativus* (domestic carrot), a novel putative psoralen synthase (CYP71AJ21) and a group II-6 member (CYP71AJ7) have been identified. Transcriptomic data from different cultivars [32] as well as PCR on *D. carota* grown in the greenhouse were used for the identification. Many of the species in tribe Daucinae are able to biosynthesize linear furanocoumarins but in small amounts compared to the species in the apioid superclade [33, 34]. *Apium graveolens* has not been described as being able to biosynthesize angular furanocoumarins. Therefore, the absence of an angelicin synthase sequence in the transcriptomic data of *A. graveolens* is not surprising.

Apioideae and Saniculoideae subfamilies (within the Apiaceae family) diverged approximately 90 million years ago from the rest of the Apiaceae family, with the *Bupleurum* genus considered being one of the earliest split outs [10]. Finding CYP71AJ subfamily members in *Bupleurum* shows that subfamily CYP71AJ has been a part of Apioideae throughout its evolutionary history.

The ancestral genes calculated for the branching points between groups I-3 and I-4 and between groups I-2 and I-3 + I-4 both resemble putative psoralen synthases. The ancestral gene for the branching point between clade II and clade I (minus subclade I-1) retains the codon coding for Trp (clade II feature) on the second position in the three-amino-acid motif, but a codon that codes for Asn (clade I feature) on the third position in the motif. The SRS4 residues coded by the codons in this ancestral genes are predicted to be the same as the ones of the members in clade II, which also applies for regions SRS5 and SRS6. For the ancestral genes between the two clades the three-amino-acid motif is the same as CYP71AJ36. The first residue pair (Leu-Asp) in SRS4 is predicted to be the same as for CYP71AJ32 and CYP71AJ36, which also applies for the second residue-pair (Val-Ala) in SRS4. All these ancestral reconstructed sequences may be too conservative in their prediction though underestimating changes that have occurred in the SRS regions. The functionality of the CYP71AJ ancestors is still a conundrum.

### Blooms of P450 genes and coevolution

Every living species is confronted at every moment with new situations to which it must adapt to live or to survive. The implementation of these evolutionary phenomena is more or less quick according to necessity. Plants have developed a large range of strategies that enable them to adapt to many situations. The strategy that is likely the most effective is related to the ability of plants to produce an arsenal of thousands of molecules deriving from universal precursors (primary metabolism) adapted to respond to many different situations. This highly complex specialized metabolism probably appears due to gene duplication phenomenon associated with neo-functionalizing mutations that result in modified substrate specificities and modifications of enzymatic reactions resulting in a blooming of enzyme families. The large number of transcriptomic reports that have been published in recent years show that the number of genes coding for cytochrome P450s is significantly higher in plants than in other organisms. For example, it may be noted that less than 10 cytochrome P450 encoding genes have been described in the baker’s yeast genome (*Saccharomyces cerevisiae*), around one hundred genes in humans and close to 250 genes have been identified in the small genome of Arabidopsis. This enzyme family therefore seems to play an important role in the adaptation of plants to their environment through their proliferation in plant genomes. In this work, we have carried out the study in a cytochrome P450 subfamily sharing more than 60 % identity with each other at the amino acid level. Heterologous expression and attempts for functional characterization allowed us to show that remodelling of the active site of these enzymes have led to changes in substrate specificity. An enlarged active site is only observed in CYP71AJ able to catalyse the synthesis of furanocoumarins, which are molecules, reported in only some apiaceous plants. It is therefore likely that these functionally characterized enzymes are deriving from older genes whose functions have not been identified yet. A broader screening geared towards smaller molecules but common to all apiaceous plants will help to unveil the functional origins of this gene family.

## Conclusion

**Fig. 5:**
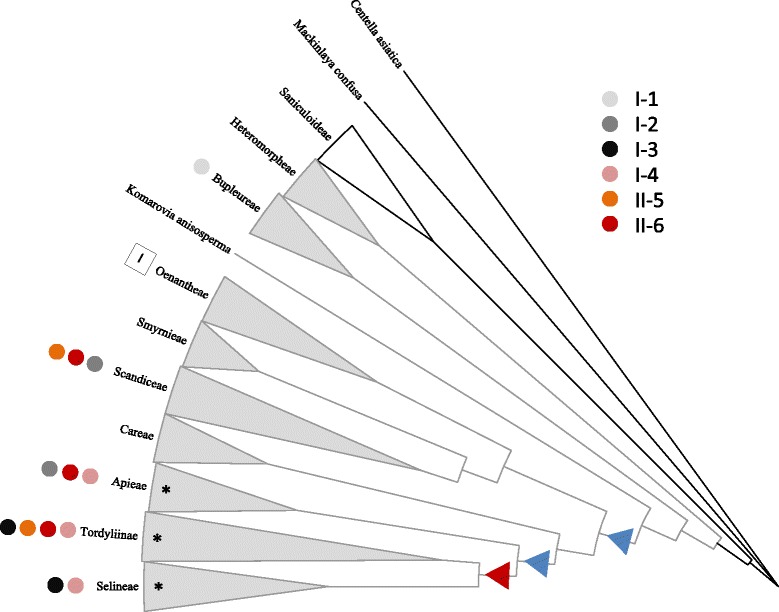
Cladogram showing the occurrence of the CYP71AJ groups across Apiaceae (tribe names are based on Downie [6, 7]). Grey-colored lineages represent the Apioideae subfamily and * denotes tribes within the apioid superclade. Red triangle: likely origin for angelicin synthase; blue triangles: potential origins for psoralen synthase. A single partial CYP71AJ has been identified in *Oenantha javaniva* (Oenantheae)

Recently, Karamat and collaborators [35] identified a single prenyltransferase from parsley able to transform umbelliferone into both demethylsuberosin and osthenol, two dehydrofuranocoumarins that are the precursors of all linear and angular furanocoumarins, respectively. Surprisingly, the same authors showed that both dehydrofuranocoumarin forms are present in plants such as parsley and *Ruta graveolens*, which do not biosynthesize angular furanocoumarins. Therefore, it seems clear that the limiting step leading to the synthesis of both kinds of isomers is related to the presence of subsequent different cytochromes P450. The biosynthesis of angular furanocoumarins is considered as a more recently evolved trait. The finding of angelicin synthases in the apioid superclade only and with the finding of putative psoralen synthases in earlier diverged lineages support this view. The angelicin synthases have likely occurred from a gene duplication of the psoralen synthase. Given that, the sequence identity between the *Angelica* (in tribe Selinae) to the *Heracleum* and *Pastinaca* (both in tribe Tordyliinae) this is likely to have happened only once (Fig. 5). The ability might have been lost in other *Angelica* spp., but this is still unresolved.

Analyses through homology modelling indicate that the size of the active sites of the clade II cytochrome P450 are smaller than the ones of clade I, with the exception of subclade I-1. The steric hindrance of substrates of these enzymes is therefore likely to be smaller. Unsuccessful incubations performed in the presence of various coumarins indicate that the substrates are not part of this group of molecules. A detailed analysis of the metabolome of some of these plants will allow us to focus on other potential target molecules.

## Methods

### Plant material

Both cultivated and collected plants used in this study were chosen in various clades (and tribes and subtribes) within Apiaceae (primarily Apioideae) and a single species within subfamily Saniculoideae. (See Additional file 2 for a list of species used in this study). The cultivated plants were grown in greenhouse at 17 °C at night and 24 °C at day and leaf materiel was collected after 5 weeks of growth. The voucher specimens are stored at the Faculty of Science herbarium, University of Copenhagen (CP), see Additional file 2 for full details. Further plant material was obtained from gardens of the Botanical Garden and Museum (Herbarium C), Natural History Museum of Denmark, University of Copenhagen. The plants grown in the greenhouse were mechanically wounded with a needle 3-4 h prior to harvesting or sprayed with Methyl Jasmonate 100 μM 48 h prior to harvesting in order to upregulate the genes coding for the biosynthesis of allelochemicals in the plants [14].

### RNA extraction

The Qiagen RNeasy Plant Mini Kit (Qiagen, The Netherlands) was used for the extraction of total RNA and cDNA was synthesized with iScript cDNA Synthesis kit (Bio-Rad, USA) as described previously [36].

### Transcriptomic data

*Pastinaca sativa* 454 transcriptomic database. Eight weeks old plantlets were sprayed with 100 μM Methyl Jasmonate and total RNA extracted 48 h post induction. The resulting RNAs were reverse transcripted, normalized and sequenced using the FLX technology by Operon MWG Biotech. The resulting singlets were assembled by Operon MWG Biotech leading to the construction of 78,117 contigs.

Transcriptomic data from other Apiaceae species were obtained from NCBI (http://www.ncbi.nlm.nih.gov): the transcriptomes for *T. garganica* and *T. laciniata* (SRA accession number SRX096991 and SRX252523, respectively) were assembled as previously described [36, 37]. The transcriptomic data (SRA accession number in bracket) for *Bupleurum chinense* (SRX080603), *Bupleurum scorzonerifolium* (SRX360424), *Apium graveolens* (DRX002997), *Petroselinum crispum* (SRX378164), *Oenanthe javanica* (SRX434017), and *Centella asiatica* (SRX257257) were assembled using CLC Genomic Workbench 7.5.1 with standard settings. The transcriptomic data from *Daucus carota* ssp *sativus* L. (domestic carrot) were also obtained from NCBI (SRP006425), and the supplementary data S2 provide an assembly of contigs that has been used here [32]. The available Apiaceae transcriptome data from the 1KP project (http://www.onekp.com/) were used for BLAST search using the different groups of CYP71AJs as templates. Samples codes are CWYJ (*Heracleum lanatum*) and TQKZ (*Angelica archangelica*).

### PCR with region-conservative primers

In order to get the full-length sequences of orthologous genes in other apiaceous species internal primer pairs were designed. For group II-6 (CYP71AJ5, CYP71AJ6, CYP71AJ7, CYP71AJ8, CYP71AJ9, CYP71AJ10, CYP71AJ11, and CYP71AJ15) orthologous, the primers pair 5’-GTAAAAAGTATTTGTGTTCTTCAGC (AJ5-int-F, forward), 5’- TGTCAATAGAAAAGGCGGAATT (AJ5-int-R, reverse) was used. For the group II-5 (CYP71AJ12, CYP71AJ13, and CYP71AJ14) orthologous the primer pair 5’-CTAACGGTGCTTCAAGCGATGAC (AJ14-int-F, forward) and 5’-CTAGATACGTGGCGTTGCAATCACCAACAG (AJ12-stop, reverse) was used. The primer pair for the II-6 was also used to obtain II-5 sequences in some cases. For putative psoralen synthases (CYP71AJ1-3 orthologous, in group I-2 and I-4) the primer pair 5’-CAAGAGGCAGATGCTGGCTC (AJ3-int-F, forward), 5’-GAGCCAGCATCTGCCTCTTG (AJ3-int-R, reverse) was used, and for the CYP71AJ4 orthologous the primer pair 5’-GGCGAGCAATTAATCCAACTAACCAG (AJ4-int-F, forward), and 5’-CCAAGGATCATATCCCAGACAATAGC (AJ4-int-R, reverse) was used. The high-fidelity *Pfu*X7 DNA polymerase was used [38] in the PCRs with a hot-start of 98 °C for 2 min followed by 30 cycles of denaturation at 98 °C for 10 s, annealing at primer specific temperatures for 20 s, and extension at 72 °C for 30s/kbp. A final 10-min extension step at 72 °C was included. In order to get the remaining sequence of the ends 5’- and 3’-RACE was performed (Clontech). Primers (see Additional file 3) were designed for the ends of the CYP71AJ genes and full-length version were obtained and validated by sequencing (Macrogen, The Netherlands). Sequences obtained from both transcriptomic data and from PCR analysis were sent to the P450 naming committee (http://drnelson.uthsc.edu/CytochromeP450.html).

### Phylogenetic analysis

Obtained sequences (either full length or partial) were aligned using default options in MUSCLE [39] as implemented in the software Geneious 6.1.8 (www.geneious.com). Phylogenetic analyses were conducted using maximum likelihood. Default options for PhyML based on the substation model LG, in Geneious 6.1.8 was chosen [40]. All maximum likelihood trees (ML) were obtained using 1000 replicates of random taxon addition sequence. All characters were included in the analyses. Clade support was assessed using non-parametric bootstrap re-sampling. Bootstrap analysis [41] was carried out using 1000 replicates. We defined bootstrap percentages (BS) < 50 % to be unsupported, between 50 % and 74 % as weak support, between 75 % and 89 % BS as moderate support, and scores of greater than 90 % BS as strong support. Alignments supporting the trees are given in Additional file 6 (for Fig. 3) and 4.

### Heterologous expression in yeast and metabolic screening

CYP71AJ5, 6, and 13 have been cloned into pYeDP60-GW. This plasmid was constructed by ligating the RfA cassette into the blunted *Bam*HI site of the original vector pYeDP60 already described by Pompon [42]. The CYP71AJ13 coding sequence has been amplified from RNA using primers with additional restriction sites and a 6xHis tag at the 3’ end (71AJ13Dir: GGATCCATGATACTTGAGCAACAACC, 71AJ13Rev: GAATTCCTA*GTGGTGATGGTGATGATG*GATACATGGCGTTGC) using Platinium®*Taq* DNA Polymerase High Fidelity (Invitrogen). The amplified product was ligated into the pCR8™/TOPO/GW (Invitrogen) as recommended and further sub-cloned into the pYeDP60-GW vector using a LR Recombinase (Invitrogen). Heterologous expression was performed in WAT11 yeast strain as previously described [13].

The enzymatic activity was determined as previously described and substrate metabolism was investigated using the described HPLC analysis [13, 43].

### dN/dS selection and residue property analyses

The 19 full-length CYP71AJ sequences were analyzed at the web interface (www.datamonkey.org) of the HyPhy software package [44] for sites under potential negative (dN/dS < 1) or positive selection (dN/dS > 1). Both MEME (Mixed Effects Model of Evolution) [26] and REL (Random Effects Likelihood) [24, 25] were used for the detection analysis using the nucleotide substitution model best describing the data (HyPhy model string: 012012). The sequences were first aligned using RevTrans v1.4 [45] based on a CLUSTAL W v1.83 alignment resulting in 509 aligned codon sites. Both methods were based on a NJ-tree. For REL the significant Bayes Factor level = 50 was chosen and for MEME a 0.05 significant-level was chosen.

PRIME (PRoperty Informed Models of Evolution) (http://hyphy.org/w/index.php/PRIME) was also used on the sequences to infer residue changes resulting in physiochemical properties that could have an impact on the activity. Five properties based on Conant-Stadler [27] and five properties based on Atchley *et al.* [28] were used and residues were selected on a corrected p-value < 0.05 level.

### Homology modeling and cavity calculations

Homology models of five CYP71AJ proteins from different groups were built using a human P450 crystal structure (CYP2A6 with an inhibitor, [PDB:2FDV.a]) as template. The template had an identity of 26 % with CYP71AJ3 (47 % positives, gaps: 4 %), 29 % with CYP71AJ4 (47 % positives, gaps: 4 %), 27 % with CYP71AJ6 (48 % positives, gaps: 4 %), 26 % with CYP71AJ12 (49 % positives, gaps: 6 %), and 27 % with CYP71AJ32 (45 % positives, gaps: 6 %). CPHmodels v3.2 (http://www.cbs.dtu.dk/services/CPHmodels/) was used for building the homology models [46]. All models had a Z-score of approximately 60, indicating high reliability models. The heme-group from the template structure was inserted in the homology model structures afterwards and covalently connected to the cysteine residue. Energy minimization was done in MOE2011.10 (Molecular Operating Environment, Chemical Computing Group Inc.) to obtain a 0.01 kcal/mol*Å gradient. Ramachandran plots were used on both minimized and non-minimized models to make sure no residues were considered illegal in the SRS regions. In order to better reflect the active sites with a potential bound substrate the non-minimized models were used for the cavity site investigations.

VOIDOO v3.3.4 (http://xray.bmc.uu.se/usf/voidoo.html) was used to calculate the sizes of the active sites in the homology models of the different CYP71AJ groups using a probe size of 1.4 Å and a mesh size of 0.3. A centre coordinate was set at 54, 76, 60, for the X-, Y-, and Z-axis, respectively.

### Ancestral sequence reconstruction

A total of 17 ancestral sequences under a MG94 model calculated from the Neilsen (2002) (‘sampled’) approach [47] with a general discrete site-to-site rate variation and 3 classes were obtained for each of the branching nodes in the strict consensus tree with CYP71AJ32 (*Bupleurum chinense*) set as the root [44], and 4 of these sequences representing splitting point between CYP71AJ subclades were used for further analysis.

## Additional files

Additional file 1:
**List of investigated coumarins.**
Additional file 2:
**Full list of all CYP71AJ members including plant material details.**
Additional file 3:
**List of primers used.**
Additional file 4:
**Figures and alignment of partial trees.**
Additional file 5:
**PRIME, MEME data.**
Additional file 6:
**Fasta of alignment for tree shown in Fig.** 2 **.**
