# Supplementary material for: Evolution of substrate recognition sites (SRSs) in cytochromes P450 from Apiaceae exemplified by the CYP71AJ subfamily
Source: BMC Evol Biol. 2015 Jun 26;15:122. doi: 10.1186/s12862-015-0396-z (PMC4482195; doi:10.1186/s12862-015-0396-z)

A. Linear furanocoumarins

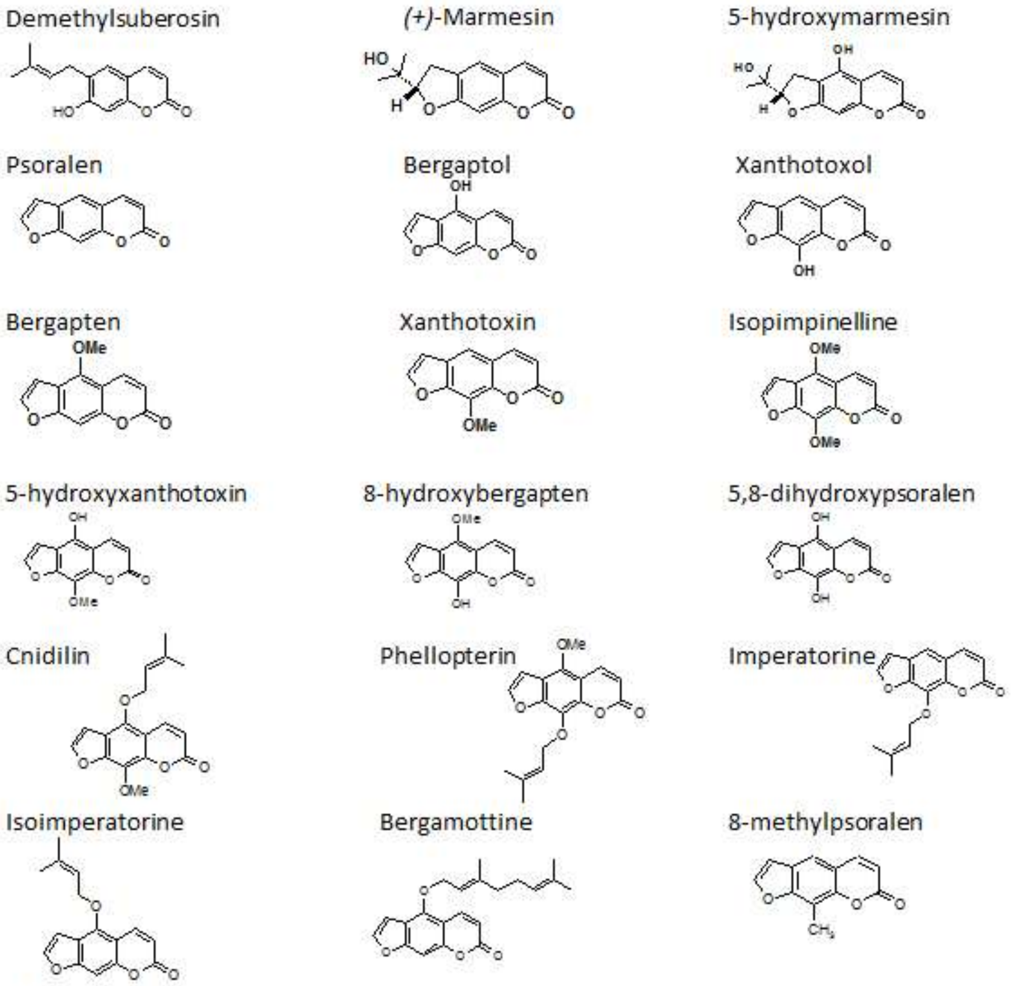

B. Angular furanocoumarins

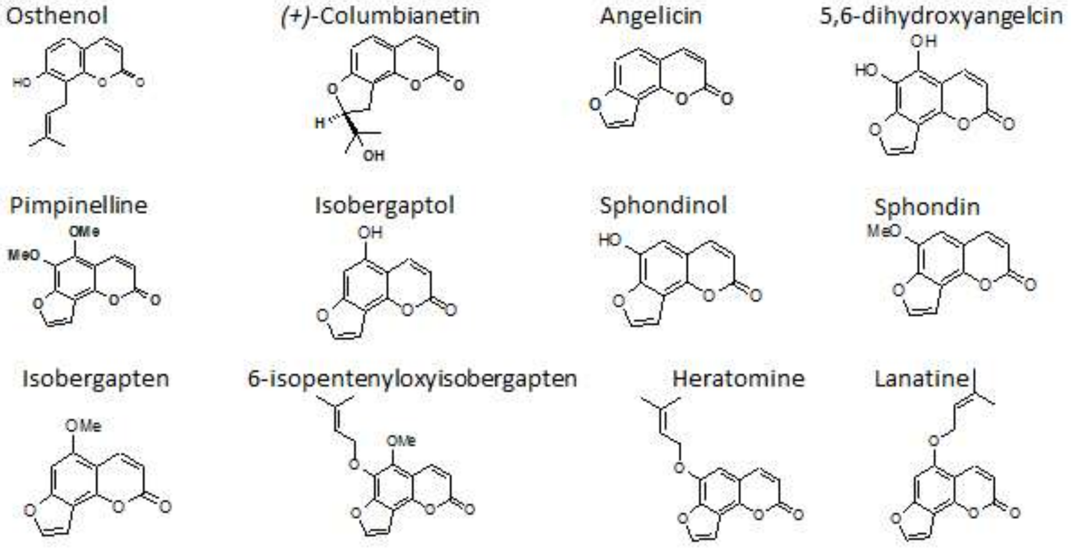

C. Coumarins

Umbelliferone

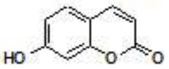

5,7-dihydroxy 4-methylcoumarin

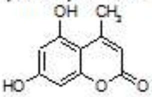

Scopoletin

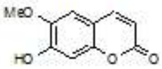

Coumarin

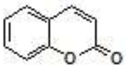

5,7-dimethoxy 3-methylcoumarin

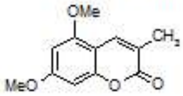

Fraxetine

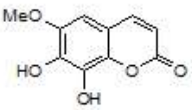

Daphnetin

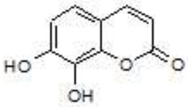

Daphnetin 7-methylether

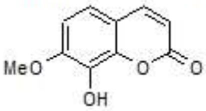

Daphnetin dimethylether

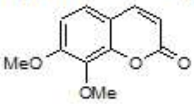

Scoparon

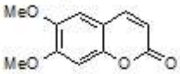

Limettine

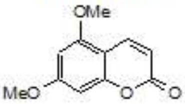

Osthol

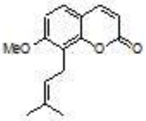

6-methoxycoumarin

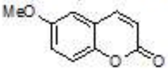

7-methoxycoumarin

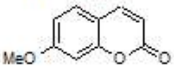

Esculetin

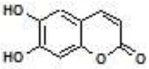

4-hydroxycoumarin

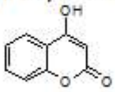

4,7-dihydroxycoumarin

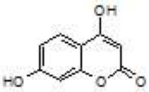

5-methylumbelliferon

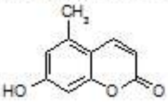

Ayapin

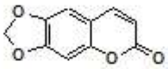

7-metthoxy 3-methylcoumarin

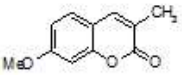

Suberosin

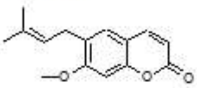

4-methyl 7-ethoxycoumarin

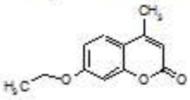

Isoscopoletin

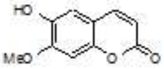

D. Smaller molecules

cinnamate

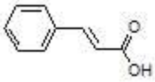

p-coumarate

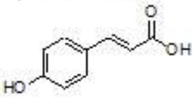

o-coumarate

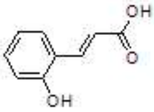

Supplement: Additional file 1: — List of investigated coumarins. [file 12862_2015_396_MOESM1_ESM.pdf]
