# Supplementary material for: Evolution of substrate recognition sites (SRSs) in cytochromes P450 from Apiaceae exemplified by the CYP71AJ subfamily
Source: BMC Evol Biol. 2015 Jun 26;15:122. doi: 10.1186/s12862-015-0396-z (PMC4482195; doi:10.1186/s12862-015-0396-z)
Supplement: Additional file 2: — Full list of all CYP71AJ members including plant material details. [file 12862_2015_396_MOESM2_ESM.docx]

## Additional file 2: List of sequences and plants material

The list gives an overview of the sequence origin. All the voucher numbers refer to Voucher Specimens stored at the Botanical Garden and Museum, Natural History Museum of Denmark, University of Copenhagen – Herbarium C

Species marked with * belong to the apioid superclade.

| Name | Species (clade) | Functionality | Full-length vs. fragment | NCBI accession number | PCR and/or transcriptome | Voucher number, contig name, or Botanical Garden ID | Location of collection | Cultivar name and seed supplier | Tissue |
| --- | --- | --- | --- | --- | --- | --- | --- | --- | --- |
| CYP71AJ1 | *Ammi majus* L. (Apieae)* | Psoralen synthase | Full-length | AY532370.2 | PCR | Larbat *et al*. (2007) |  | Wild | Leaf |
| CYP71AJ2 | *Apium graveolens* L. (Apieae)* | Psoralen synthase | Full-length | EF191022.1 | PCR | Larbat *et al*. (2009) |  | Cultivated | Leaf |
| CYP71AJ3 | *Pastinaca sativa* L. (Tordyliinae, Tordylieae)* | Psoralen synthase | Full-length | EF191020.1 | PCR | Larbat *et al.* (2009) |  | Cultivated | Leaf |
| CYP71AJ4 | *Pastinaca sativa* L. (Tordyliinae, Tordylieae)* | Angelicin synthase | Full-length | EF191021.1 | PCR | Larbat *et al*. (2009) |  | Cultivated | Leaf |
| CYP71AJ5 | *Thapsia garganica* L. (Daucinae, Scandiceae) | Unknown | Full-length | KP191555 | Transcriptome and PCR | HTS 2010-13  Science, Uni. Copenhagen (CP) | Italy, 40.898625N, 16.706139E | Wild | Root, fruit |
| CYP71AJ6 | *Thapsia laciniata* Rouy, (Daucinae, Scandiceae) | Unknown | Full-length | KP191554 | Transcriptome and PCR | HTS 2010-01  Science, Uni. Copenhagen (CP) | France, 43.540958N, 6.816158E | Wild | Root |
| CYP71AJ7 | *Daucus carota* ssp *sativus* (Daucinae, Scandiceae) | Unknown | Full-length | KP191557 | PCR | HTS 2013-04  SNM, Uni. Copenhagen (C) |  | Cultivar: Cubic  Seeds from: froetorvet.dk | Leaf |
| CYP71AJ8 | *Ammi majus* L. (Apieae)* | Unknown | Full-length | KP191553 | PCR | N/A | Germany | Wild | Leaf |
| CYP71AJ9 | *Petroselinum crispum* Mill (Apieae)* | Unknown | Full-length | KP191552 | PCR | HTS 2013-01  SNM, Uni. Copenhagen (C) |  | Culitvar: comun 3.  Seeds from: froetorvet.dk | Leaf |
| CYP71AJ10 | *Pastinaca sativa* L. (Tordyliinae, Tordylieae)* | Unknown | Partial (132-295) |  | PCR | HTS 2013-03  SNM, Uni. Copenhagen (C) |  | Cultivar: White gem  Seeds from: froetorvet.dk | Leaf |
| CYP71AJ11 | *Heracleum mantegazzium* L. (Tordyliinae, Tordylieae)* | Unknown | Full-length | KP191551 | PCR | HTS 2010-16  Science, Uni. Copenhagen (CP) | Denmark, 55.766203N, 12.459442E | Wild | Leaf |
| CYP71AJ12 | *Thapsia laciniata* Rouy, (Daucinae, Scandiceae) | Unknown | Full-length | KP191559 | Transcriptome and PCR | HTS 2010-01  Science, Uni. Copenhagen (CP) | France, 43.540958N, 6.816158E | Wild | Root |
| CYP71AJ13 | *Pastinaca sativa* L. (Tordyliinae, Tordylieae)* | Unknown | Full-length | KP191560 | Transcriptome and PCR | HTS 2013-03  SNM, Uni. Copenhagen (C) |  | Cultivar: White gem  Seeds from: froetorvet.dk | Leaf |
| CYP71AJ14 | *Thapsia garganica* L. (Daucinae, Scandiceae) | Unknown | Full-length | KP191558 | Transcriptome and PCR | HTS 2010-13  Science, Uni. Copenhagen (CP) | Italy, 40.898625N, 16.706139E | Wild | Root, fruit |
| CYP71AJ15 | *Laserpitium siler* L. (Daucinae, Scandiceae) | Unknown | Full-length | KP191556 | PCR | HTS 2010-09  Science, Uni. Copenhagen (CP) | Italy, 42.69721N, 13.622655E | Wild | Root |
| CYP71AJ16 | *Thapsia garganica* **L.** (Daucinae, Scandiceae) | Unknown | Partial (43-272) |  | Transcriptome and PCR | HTS 2010-13  Science, Uni. Copenhagen (CP) | Italy, 40.898625N, 16.706139E | Wild | Fruit |
| CYP71AJ17 | *Anthricus sylvestris* Hoffm. (Scandicinae, Scandiceae) | Unknown | Partial (17-295) |  | PCR | HTS 2010-05  Science, Uni. Copenhagen (CP) | Italy, 43.252017N, 12.800360E | Wild | Leaf |
| CYP71AJ18 | *Peucedanum cervaria* L. (Selineae)* | Unknown | Partial (146-248) |  | PCR | HTS 2010-04  Science, Uni. Copenhagen (CP) | Italy, 43.252017N, 12.800360E | Wild | Leaf |
| CYP71AJ19 | *Seseli montanum* L. (Selineae)* | Putative psoralen synthase | Partial (182-399) |  | PCR | P1972-5024 |  | Botanical Garden | Leaf |
| CYP71AJ20 | *Angelica archangelica* ssp *archangelica* L. (Selineae)* | Putative psoralen synthase | Near full-length (16-482) |  | PCR | E4456-0006B |  | Botanical Garden | Leaf |
| CYP71AJ21 | *Daucus carota* ssp *sativus* (Daucinae, Scandiceae) | Putative psoralen synthase | Full-length | KP191562 | Transcriptome and PCR | HTS 2013-04  Science, Uni. Copenhagen (CP) |  | Cultivar: Cubic  Seeds from: froetorvet.dk and from SRP006425, SRA, NCBI | Leaf |
| CYP71AJ22 | *Haracleum mantegazzianum* L. (Tordyliinae, Tordylieae)* | Putative angelicin synthase | Near full-length (16-488) |  | PCR | HTS 2010-16  Science, Uni. Copenhagen (CP) | Denmark, 55.766203N, 12.459442E | Wild | Leaf |
| CYP71AJ23 | *Angelica archangelica* ssp *archangelica* L. (Selineae)* | Putative angelicin synthase | Partial (196-341) |  | PCR | E4456-0006B |  | Botanical Garden | Leaf |
| CYP71AJ24 | *Ferula communis* ssp *glaucus* (Ferulinae, Scandiceae) | Unknown | Partial (199-291) |  | PCR | HTS 2010-08  Science, Uni. Copenhagen (CP) | Italy, 43.177353N, 12.996022E | Wild | Leaf |
| CYP71AJ25 | *Thapsia garganica* L. (Daucinae, Scandiceae) | Putative psoralen synthase | Partial (293-473) | KP191563 | Transcriptome | HTS 2010-13  Science, Uni. Copenhagen (CP) | Italy, 40.898625N, 16.706139E | Wild | Fruit |
| CYP71AJ26 | *Heracleum lanatum* Bertram. (Tordyliinae, Torlylieae)* | Unknown | Partial (1-409) |  | Transcriptome | Contig 2002479 |  | Sample name: CWYJ  From: www.onekp.com | Flower, stem, leaf |
| CYP71AJ27 | *Heracleum lanatum* Bertram. (Tordyliinae, Torlylieae)* | Putative psoralen synthase | Full-length |  | Transcriptome | Contig 2002478 |  | Sample name: CWYJ  From: www.onekp.com | Flower, stem, leaf |
| CYP71AJ28 | *Heracleum lanatum* Bertram. (Tordyliinae, Torlylieae)* | Putative angelicin synthase | Near full-length (1-40, 57-500) |  | Transcriptome | Contig 2002477 |  | Sample name: CWYJ  From: www.onekp.com | Flower, stem, leaf |
| CYP71AJ29 | *Heracleum lanatum* Bertram. (Tordyliinae, Torlylieae)* | Unknown | Near full-length (9-494) |  | Transcriptome | Contig 2011531 |  | Sample name: CWYJ  From: www.onekp.com | Flower, stem, leaf |
| CYP71AJ30 | *Heracleum lanatum* Bertram. (Tordyliinae, Torlylieae)* | Putative psoralen synthase paralog | Partial (1-354) |  | Transcriptome | Contig 2002677 |  | Sample name: CWYJ  From: www.onekp.com | Flower, stem, leaf |
| CYP71AJ31 | *Bupleurum chinense* L. (Bupleureae) | Unknown | Partial (376-503) |  | Transcriptome |  |  | SRX080603  From SRA, NCBI | Root |
| CYP71AJ32 | *Bupleurum chinense* L. (Bupleureae) | Unknown | Full-length |  | Transcriptome |  |  | SRX080603  From SRA, NCBI | Root |
| CYP71AJ33 | *Bupleurum salicifolium* R.Br. (Bupleureae) | Unknown |  |  | PCR | S2006-0495A |  | Botanical Garden | Leaf |
| CYP71AJ35 | *Pastinaca sativa* L. (Tordyliinae, Tordylieae)* | Unknown | Partial (299-494) |  | Transcriptome | Contig 10479 |  | Cultivated | Leaf |
| CYP71AJ36 | *Pastinaca sativa* L. (Tordyliinae, Tordylieae)* | Unknown | Partial (302-494) |  | Transcriptome | Contig 29131 |  | Cultivated | Leaf |
| CYP71AJ37 | *Pastinaca sativa* L. (Tordyliinae, Tordylieae)* | Unknown | Partial (1-145) |  | Transcriptome | Contig 61375 |  | Cultivated | Leaf |
| CYP71AJ38 | *Oenanthe javanica* (Blume) DC. | Unknown | Partial (76-424) |  | Transcriptome | Contig 14855 |  | SRX434017  From SRA, NCBI | General |
| CYP71AJ39 | *Bupleurum scorzonerifolium* L. | Unknown | Partial (43-297) |  | Transcriptome | Contig 4488 |  | SRX360424  From SRA, NCBI | Root |
| CYP71AJ40 | *Apium graveolens* L. (Apieae)* | Unknown | Full-length |  | Transcriptome | Contig 16543 |  | SRX326597  From SRA, NCBI | Whole plant |
