# Supplementary material for: Evolution of substrate recognition sites (SRSs) in cytochromes P450 from Apiaceae exemplified by the CYP71AJ subfamily
Source: BMC Evol Biol. 2015 Jun 26;15:122. doi: 10.1186/s12862-015-0396-z (PMC4482195; doi:10.1186/s12862-015-0396-z)
Supplement: Additional file 3: — List of primers used. [file 12862_2015_396_MOESM3_ESM.pdf]

Additional file 3

List of primers used for PCR-based fishing approach.

Primer name	Sequence (5'-3') (F/R)	Targets (perfect seq match)	Tm (°C)
AJ5-int-F	GTAAAAAGTATTTGTGTTCTTCAGC (F)	CYP71AJ5, CYP71AJ6, CYP71AJ12, CYP71AJ14	52
AJ5-int-R	TGTCAATAGAAAAGGCGGAATT (R)	CYP71AJ5, CYP71AJ7, CYP71AJ15, CYP71AJ17, CYP71AJ18	54
AJ5-ATG	ATGATGATGGACCAGCAAACCC (F)	CYP71AJ5	59
AJ5-stop	CTAGACTCGTGATGTCGCAATC (R)	CYP71AJ5, CYP71AJ6	56
AJ6-ATG	ATGATGGACCAGCAAACCCTGTTTCTATCT (F)	CYP71AJ6	62
AJ7-F	ATGATGGACCAACAAGCCTTG (F)	CYP71AJ7	57
AJ7-R	CTAGACTCGTGATGTCGCAAT (R)	CYP71AJ7	56
AJ8-F	ATGGACCAACAATCCTTGTTTC (F)	CYP71AJ8	55
AJ8-AJ9-R	TTAGACACGTGATGTTGCGA (R)	CYP71AJ8, CYP71AJ9	55
AJ12-ATG	ATGATACTAGATCAACAACTCTTGTTTCTGTC (F)	CYP71AJ12	57
AJ12-stop	CTAGATACGTGGCGTTGCAATCACCAACAG (R)	CYP71AJ12, CYP71AJ14	64
AJ14-ATG	ATGATACTAGATCAACAATTCTTGTTTCTATC (F)	CYP71AJ14	54
Bc_red-R	TCAAACATGTGGTTTGGCAATCACCAAAAG (R)	CYP71AJ31	59
Bc_blue-F	ATGAAGGACCAATATCTTCTCTACCTATATTTC (F)	CYP71AJ32	58
Bc_green-R	CCTGTGAGTAAATCAACCCAACAAAGCAA (R)	CYP71AJ32	59
