# Supplementary material for: Evolution of substrate recognition sites (SRSs) in cytochromes P450 from Apiaceae exemplified by the CYP71AJ subfamily
Source: BMC Evol Biol. 2015 Jun 26;15:122. doi: 10.1186/s12862-015-0396-z (PMC4482195; doi:10.1186/s12862-015-0396-z)
Supplement: Additional file 4: — Figures and alignment of partial trees. [file 12862_2015_396_MOESM4_ESM.pdf]

Additional file 4

Partial tree of the selection of AA 120-220 from the alignment made for figure 2. Please observe, that the clade marking is based on Figure 2, and not all groups and clades are well supported in this tree.

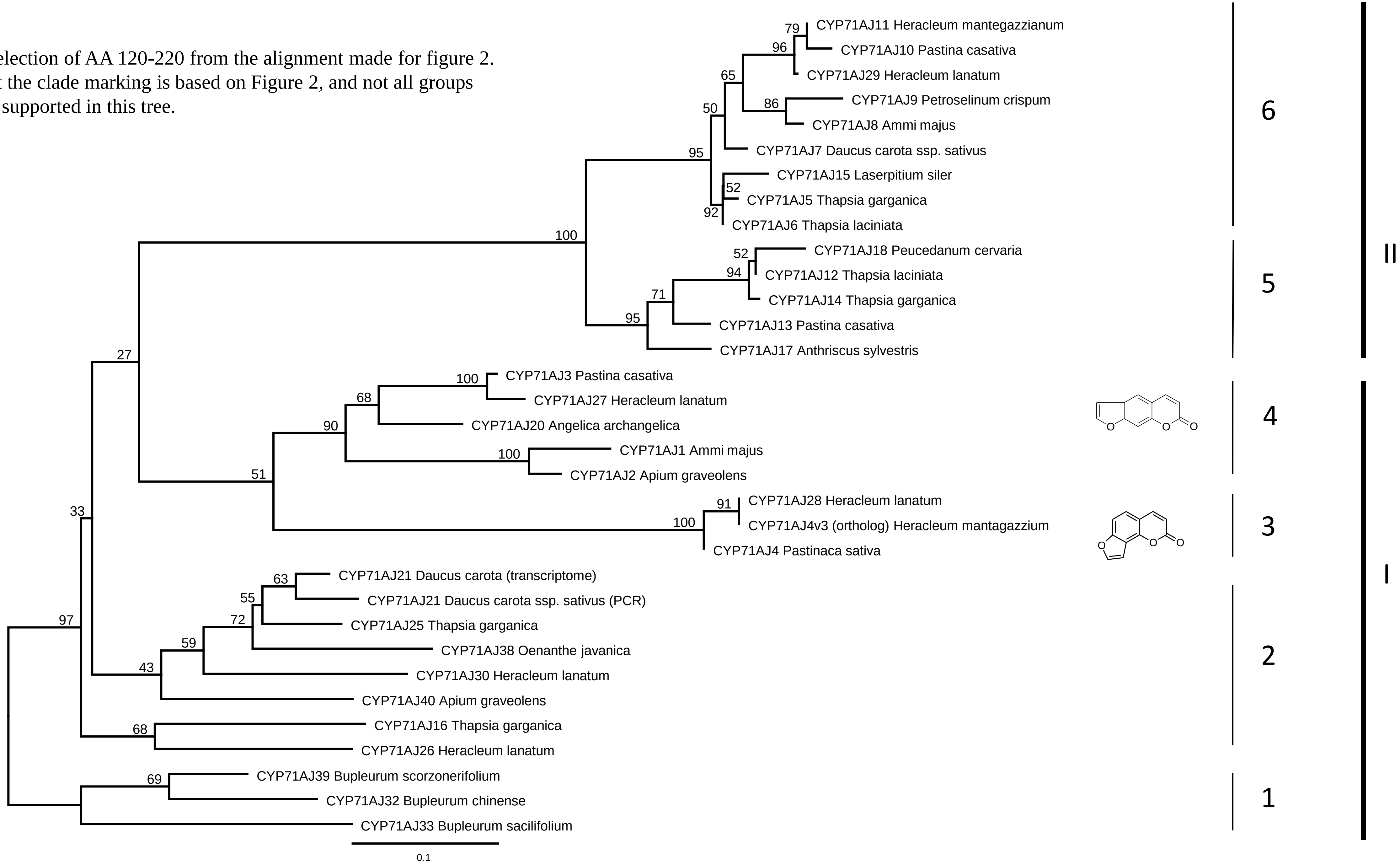

Muscle Alignment of partial sequences from AA 120-220

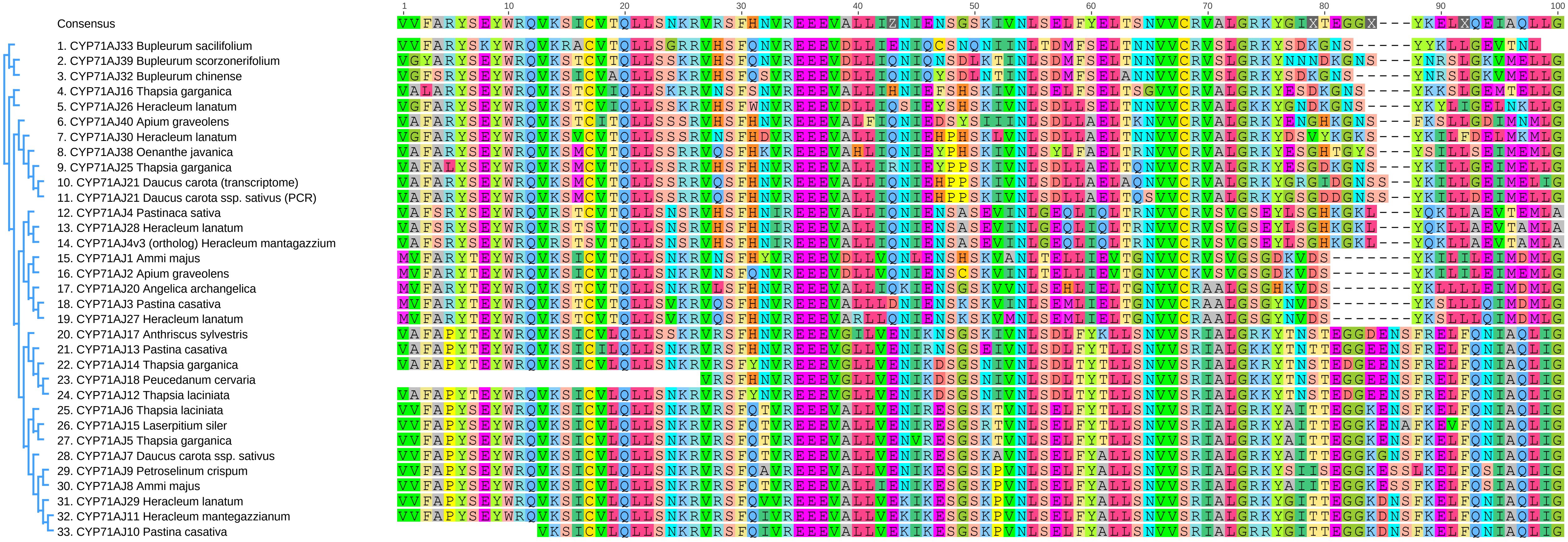

Supplementary file 5
Partial tree of the selection of AA 203-307 from the alignment made for figure 2.
Please observe, that the clade marking is based on Figure 2, and only a few groups and clades are well supported in this tree.

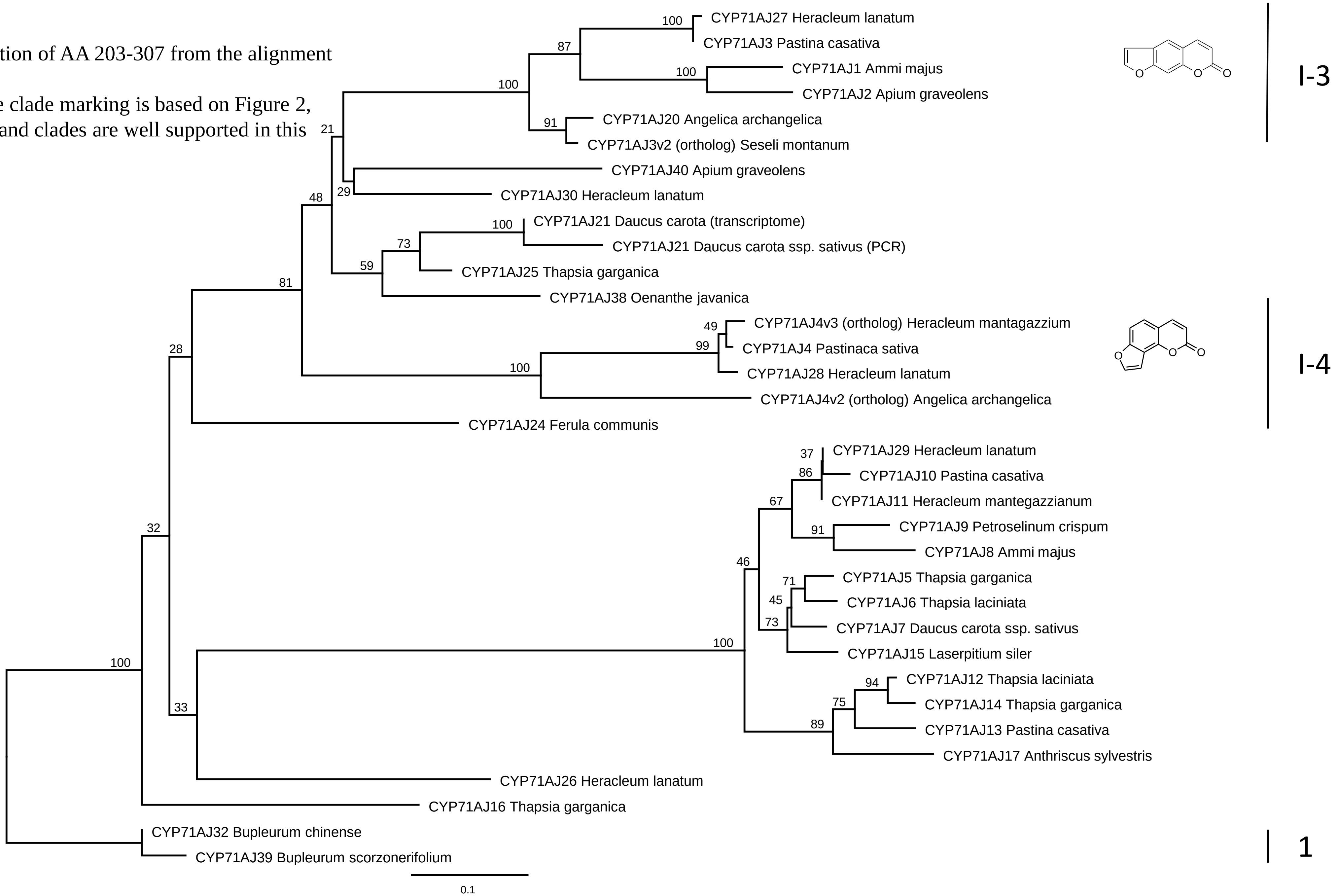

Muscle Alignment of partial sequences from AA 203-307

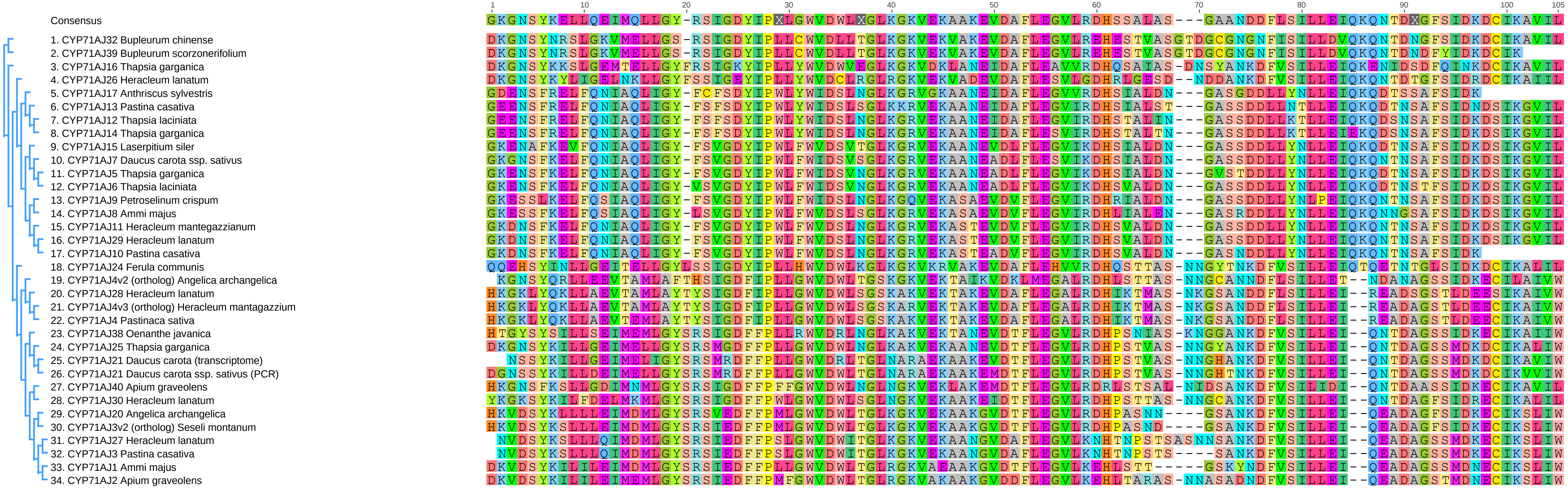

Supplementary file 5
Partial tree of the selection of AA 380-509 from the alignment made for figure 2.
Please observe, that the clade marking is based on Figure 2, and not all groups and clades are well supported in this tree.

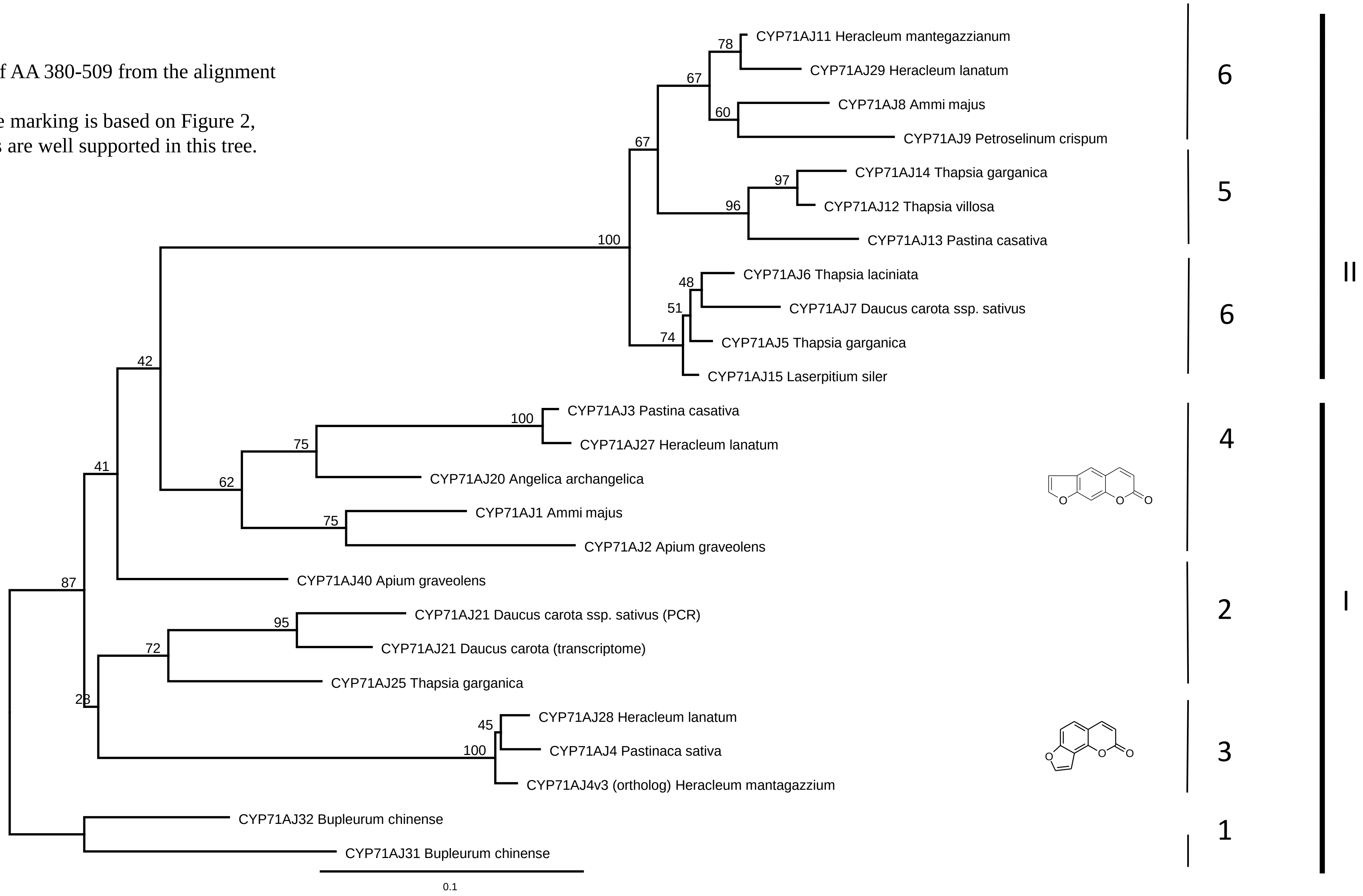

Muscle Alignment of partial sequences from AA 380-509

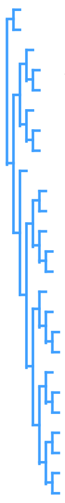

Sequence	1–128
Consensus	PREAXQDVKVMGYDIKAGTQVLINAWAIARDPSLWDNPEEFRPERFLNSPIDYKGLHYEFIPFGAGRRGCPGIQFAMAVNELALANLVHKFDFXLPDGKRLEDLDMTAETGMTLHKKSPLLVIATPHV
1. CYP71AJ32 Bupleurum chinense	PREARKDVKVMGYDIRSGTQVIINAWAIARDPSLWNNPEEFRPERFLDSPIDYKGVHYEFIPFGGGRRMCPGIQFAMAVNELAIANIVHKFDFELPDGERLQDLDMTCVTGVTLCRKSPLLVIAKPHV
2. CYP71AJ31 Bupleurum chinense	PREARNDVNVMGYDISSGTQVL-NSWAIARDPSLWNNPDEFRPERFLNSPIDYKGVHYEYLPFGGGRRMCPGIQFAVAVNELAIANIVHKFNFELPDGQRLEDLDMTGVTGISVCRKSPLLVIAKPHV
3. CYP71AJ25 Thapsia garganica	PREARQDVKVMGYDIRKGTQVLINAWAIARDPSLWENPEKFQPERFLNSPIDYKGLHYEYLPFGGGRRGCPGIQFAMAVNELAVANVVHKFDFELPNGETREDLDMTGVTGITLRRKSPLLVIATPHV
4. CYP71AJ21 Daucus carota ssp. sativus (PCR)	PREARQDVKVMGYDIRKGTQLVINAWAIARDPSLWDNPEEFRPERFLNSPIDYKGLHYDYLPFGAGRRGCPGYQFAMAVNELALANVVNKFDFELPNGERKEDLDMAGVNGLTL
5. CYP71AJ21 Daucus carota (transcriptome)	PREARQDVKVMGYDIRKGTQLVINAWAIARDPSLWDSPEEFRPERFLNSPIDYKGLHYEYLPFGAGRRGCPGYHFAMAVNELALANVVHKFDFELPNGERKEDLDMTGVTGLTVRRKSPLLVIATPHV
6. CYP71AJ4v3 (ortholog) Heracleum mantagazzium	PREAKQDVKLMGYDISSGTQVLINTWETARDPSLWDNPEEFRPERFFNSPIDYKGLHYEYLPFGGGRRGCPGIQFAMAVNELAVANVVYKFDFKMPDGERFEDLDMSGVPGISLSK
7. CYP71AJ28 Heracleum lanatum	PREAKQDVKLMGYDISSGTQVLINTWETARDPSLWDNPEEFRPERFFNSPIDYKGLHYEYLPFGGGRRGCPGIQFAMAVNELAVANVVYKFDFKMPDGERFEDLDMSGVPGISLYRKYPLLVVATPHV
8. CYP71AJ4 Pastinaca sativa	PREAKQDVKLMGYDISSGTQVLINTWETARDPSLWDNPEEFRPERFLNSPIDYKGLHYEYLPFGGGRRGCPGIQFAMAVNELAVANVVYKFDFKMPDGERFEDLDMSGVPGISLYRKYPLMVVASPHV
9. CYP71AJ40 Apium graveolens	PRQARQDVKVMGYDIRSGTQVLINAWAIARDPSLWDKPEEFRPERFLNSPIDYKGLHYEYVPFGAGRRGCPGIQFAMAVNELAVANVVHKFDFKLPDGKRLEDLDMTAVTGLTLRRESPLLVVATPHI
10. CYP71AJ1 Ammi majus	PREARQDIKFMGYDISSGTQVLINAWAIARDPLLWDKPEEFRPERFLNSPIDYKGFHYEFLPFGAGRRGCPGIQFAMCINELVVANLVHKFNFELPDGKRLEDLDMTAASGITLRKKSPLLVVARPHV
11. CYP71AJ2 Apium graveolens	PRESRQDVKFMGYDISAGTQVLINVWAIARDPSLWEKPEEFRPERFLNSHIDYKGFNYEYLPFGAGRRGCPGIQFAMAVNELVVANVIHKFNFELPDGERLEDLDMTAVSGITLRKKSPLLVVARPHV
12. CYP71AJ20 Angelica archangelica	PRETRQDVKFMGYDIKAGTQVLINAWAIARDPSLWDNPEEFRPERFLNTPIDYKGFHYEYLPFGAGRRGCPGIQFAISVNELAVANVVHKFNFELPDGKRLEDLDMTGSTGITLSKK
13. CYP71AJ3 Pastina casativa	PREARQDVKFMGYDIKSGTQVLINAWAIARDPSSWDNPEEFRPERFLNSPIDYKGFNYEYIPFGAGRRGCPGIQFAISVNELVVANVVNKFNFELPDGKRLEEMDMTASTGITFHKKSPIFVVATPHV
14. CYP71AJ27 Heracleum lanatum	PREARQDVKFMGYDIKSGTQVLINAWAIARDPASWDNPEEFLPERFLNSPIDYKGFNYEYIPFGAGRRGCPGIQFAISVNELVVANVVNKFNFELPDGKRLEEMDMTASTGITFHKKSPIFVVATPHV
15. CYP71AJ15 Laserpitium siler	AREAMQDVKVMGYDVKAGTQVLINAWAIATDPALWDNPEEFIPERFLNNPTDYKGLHFEFIPFGAGRRGCPGIQYAMAINELALANLVHIFDFALPDGRRLEDLDMTSETGMTLHKKSPLLVIATSRV
16. CYP71AJ5 Thapsia garganica	AREAMQDVKVMGYDVKAGTQVLINAWAIATDPALWDNPEKFIPERFLNNPIDYKGLHFEFIPFGAGRRGCPGIQYAMAINELALANLVHIFDFALPDGRRLEDLDMTSETGMTLHKKSPLLVIATSRV
17. CYP71AJ6 Thapsia laciniata	AREAMQDVKLMGYDVKSGTQVLINAWAIATDPALWDNPEEFIPERFLNNPIDYKGLHFEFIPFGAGRRGCPGIQYAMAINELALANLVHIFDFALPDGRRLEDLDMTSETGMTLHKKSPLLVIATSRV
18. CYP71AJ7 Daucus carota ssp. sativus	AREAMQDVKVMGYDVKDGTQVLINAWAIATDPALWDNPEEFIPERFLNNPIDYKGLHFEFIPFGAGRRGCPGIQYAMAINELAVANLVHIFDFALPDGRRLEDLDLTSETGMTLHKKSPLMVIATSRV
19. CYP71AJ13 Pastina casativa	AREAMQDVKVMGYDIKAGTQVLINAWAIATDGAVWDNPEEFIPERFLNNPIDYKGLHFEFIPFGAGRRGCPGIQYAMAINELALANLVHIFDLALPDGKRFENLDMDSETGMTVHKKSPLLVIATPCI
20. CYP71AJ14 Thapsia garganica	AREAMQDVKVMGYDIKAGTQVLINAWAIATDPAVWDNPGEFIPERFLNNSVDYKGLHFEFIPFGAGRRGCPGIQYAMAINELALATLVHIFDFALPDGKRFEDLDMASETGMTVHKKSPLLVIATPRI
21. CYP71AJ12 Thapsia villosa	AREAMQDVKVMGYDIKAGTQVLINAWAIATDPAVWDNPEEFIPERFLNNSVDYKGVHFEFIPFGAGRRGCPGIQYAMAINELALANLVHIFDFALPDGKRFEDLDMASETGMTVHKKSPLLVIATPRI
22. CYP71AJ11 Heracleum mantegazzianum	AREAMQDVNVMGYDIKAGTQVLINAWAIATDPTLWDNPEEFIPERFLNNPVDYKGLHFEFIPFGAGRRGCPGIQYAMAINELALANLVHIFDFALPDGKRFEDLDMTAETGMTLHKKSPLLAIATSRV
23. CYP71AJ29 Heracleum lanatum	AREAMQDVNVMGYDIKAGTQVLINAWAIATDPTLWDNPEEFIPERFLNNPVDYKGLHFEFIPFGAGRRGCPGIQYAMAINELALANLVHIFDFTLPDGKRFEDLDMAAETGMTLHKKSPLLAIATSCV
24. CYP71AJ8 Ammi majus	AREAMQDVNVMGYDIRAGTQVMINAWAIATDPTVWDKPEEFIPERFLNNPLDYKGLHFEFIPFGAGRRGCPGIQYAMAINELALANLVHIFDFALPDGKRFEDLDMDAETGMTLHKKSPLLVIATSRV
25. CYP71AJ9 Petroselinum crispum	AREAMQDVNVMGYYIKAGTQVMINAWAIATDPTLWDNPEEFIPERFLNNPVDHKGMHFEFIPFGARRRGCPGIQYAMAINELALANLVHIFDFALPGGKRLEDLDMDAETGMTLHKKSPLLVVATSRV
