# Supplementary material for: Evolution of substrate recognition sites (SRSs) in cytochromes P450 from Apiaceae exemplified by the CYP71AJ subfamily
Source: BMC Evol Biol. 2015 Jun 26;15:122. doi: 10.1186/s12862-015-0396-z (PMC4482195; doi:10.1186/s12862-015-0396-z)
Supplement: Additional file 6: — Fasta of alignment for tree shown in Fig. 2 . [file 12862_2015_396_MOESM6_ESM.docx]

Fasta Alignment view of tree shown in Figure 2:

1 10 20 30 40 50 60

| | | | | | |

CYP71AV1 A. annua ------MALSLTTSIALATILLFVYKFATRSKSTKKSL-PEPWRLPIIGHMHHLIGTTPH

CYP71A1 S. melongena --MDVPCLWYSLLILLLLFIFLLIHHCFTTSKTQNMFLPPSPRKLPIIGNLHQ-LGSHPH

CYP71AJ14 T.garganica -MILDQQFLFLSLCSL-FFVLVFLYIWLSTTKTTGKNVPPSPRKLPIIGNLHL-VNQDPH

CYP71AJ12 T.laciniata -MILDQQLLFLSLCSL-FFVLVFLYIWLSTTKTTGKNVPPSPRKLPIIGNLHQ-VNQDPH

CYP71AJ13 P.sativa -MILEQQPLFLSICSL-LFVLVFLYLWLSTSRTTGKNLPPSPPKLPIIGNLHQ-VNQDPH

CYP71AJ6 T.laciniata --MMDQQTLFLSLCSM-LSVLVFLYIWLSTSKTTGKNLPPSPQKLPIIGNLHQ-VNQDPH

CYP71AJ5 T.garganica -MMMDQQTLFLSLCSM-FFVLVFLYIWLSTSKTTGKNLPPSPQKLPIIGNLHQ-VNQDPH

CYP71AJ15 L.siler --MMDQQALFLSLCSM-FFVLGFLYIWLSTSKTTGKNLPPSPQKLPIIGNLHQ-VNQDPH

CYP71AJ7 D.carota --MMDQQALFLSLSLM-FFVLAFLYIWLSASKTSSKNLPPSPGKLPIIGNLHQ-VNQDPH

CYP71AJ9 P.crispum MVLDQQSLFLLSLCSL-FFVLVFLYIWLSTSKPTGKNLPPSPRKLPIIGNLHQ-VNKDPH

CYP71AJ11 H.mantegazzium ---MDQQSLFLCLCFL-FFVLVFLYIWLSTSKTTGKNLPPSPRKLPIIGNLHQ-VNQDPH

CYP71AJ8 A.majus ---MDQQSLFLSLCSL-FFVLVFLYIWLSTSKPTGKNLPPSPRKLPIIGNLHQ-VNKDPH

CYP71AJ3 P.sativa MKMLEQNPLYLYFFPL-FLVTIFLYKWL-VKKTPSKNLPPSPPRLPIIGNLHQ-IGPDPQ

CYP71AJ27 H.lanatum MKMLEQNPLYLYFFSL-FLVTIFLYKWL-VKKTPSKNLPPSPPKLPIIGNLHQ-IGPDPQ

CYP71AJ1 A.majus MKMLEQNPQYLYFFSL-FLVTIFLYKWLTLKKTPLKNLPPSPPQYPIIGNLHQ-IGPDPQ

CYP71AJ2 A.graveolens MKMLEQNPQYLYFFSL-FLVTVFVYKLLTLKKTPSKNLPPSPPRYPIIGNLHQ-IGPDPQ

CYP71AJ4 P.sativa MKMLEQNPLYLYFFSL-FLVTIFLYKWL-AKKTPSKNLPPSPPRLPIIGNLHQ-IGPDLH

CYP71AJ21 D.carota MKMVEQYPLYLYFFSL-LSATIFFYKWLTLKKTALRNLPPSPPRFPVIGNLHQ-VGPDPY

CYP71AJ25 T.garganica MKMLEQYHLYVYFFSL-ISATIFLYKWLTLRKTALKNLPPSPPTFPIIGNLHQ-IGPDPH

CYP71AJ40 A.graveolens --MPEQYPLYLYPFSL-FLVTILLYNWLTHRKLVLEKLPPSPPKFPIIGNLHQ-IGPDPH

CYP71AJ32 B.chinensis --MKDQYLLYLYFFLISFITLIFLYKWFLNTKAALKNLPPSPPKLPIIGNLHQ-LGQDPH

CYP71AV1 A. annua RGVRDLARKYGSLMHLQLGEVPTIVVSSPKWAKEILTTYDITFANRPETLTGEIVLYHNT

CYP71A1 S. melongena RSLRKLSQKYGPVMLLHLGSKPVIVASSVDAARDILKTHDHVWATRPKYSIADSLLYGSK

CYP71AJ14 T.garganica VALRSLAQKYGPFMLLHLGSVPVLVVSSADAAKEIMKTHDLAFANRPVSSIWSRIFYNGK

CYP71AJ12 T.laciniata VALRSLAQKYGPFMLLHFGSVPVLVVSSADAAKEIMKTHDLAFANRPDSSIWSRIFYNGK

CYP71AJ13 P.sativa VALRSLAQKYGPVMQLHFGSVPVLVVSSADAAKEIMKTHDLAFANRPDSSIWSRIFYNGK

CYP71AJ6 T.laciniata ISLRSLAKKYGPVMQLHFGSIPVLVVSSADAAKEIMKTHDLAFANRPNSSIWDKIFYNGK

CYP71AJ5 T.garganica ISLRSLAKKYGPVMQLHFGSIPVLVVSSADAAMEIMKTHDLAFANRPNSSIWDKIFYNGK

CYP71AJ15 L.siler ISLRSLAKKYGPVMQLQFGSIPVLVVSSADAAKEIMKTHDLAFANRPNSSIWDRIFYNGK

CYP71AJ7 D.carota IALRSLAKKYGPVMQLQFGSVPVLVVSSADAAKEVMKTHDLAFANRPNSSIWDRIFYNGK

CYP71AJ9 P.crispum ISLRSLAKRYGQIMQLHFGSVPVLVVSSADAAKEIMKTHDLAFANRPNSSIWDRIFYKGK

CYP71AJ11 H.mantegazzium ISLRSLAKRYGPVMQLHFGSVPVLVVSSADAAKEIMKTHDLAFANRPNSSIWDGIFYNGK

CYP71AJ8 A.majus ISLRSLAKRYGPLMQLHFGSVPVLVVSSADAAKEIMKTHDLAFANRPNSSIWDSIFYKGK

CYP71AJ3 P.sativa ISLRDLAREYGPVMHLKFGSVPVLVVSSADGAREIFKTHDLVFADRPYSSVANRIFYNGR

CYP71AJ27 H.lanatum ISLRDLARKYGPVMHLKFGSVPVLVVSSADGAREIFKTHDLVFADRPYSSVANRIFYNGR

CYP71AJ1 A.majus ASLRDLAQKYGPLMFLKFGTVPVLVVSSADAAREALKTHDLVFADRPYSSVANKIFYNGK

CYP71AJ2 A.graveolens HSLRDLALKYGPLMSLKFGTVPVLVVSSADAAREVLKTHDLIFADRPYSSVANKVFYNGK

CYP71AJ4 P.sativa ISLRDLARKYGPLMQLQLGRIPVLVVSSAEATREVLKTHDVVFSQRPITSAIDKLCYKGR

CYP71AJ21 D.carota ISLRTLAEKYGPLMLLKFGSVPVVVVSSAEAAREILKTHDLVFADRPFLSVANRIFYKGR

CYP71AJ25 T.garganica ISLRALAEKFGPLMLLKFGSVPVLVVSSADAAREILKTHDLVFSDRPISSVANRLFYNGR

CYP71AJ40 A.graveolens ISLRALAQRYGPLKLLKFGRVPVLVVSSADAAREILKTHDLVFSDRPSSSVSNRIFYNGR

CYP71AJ32 B.chinensis IILKSLAQRYGPLMLLKFGSVPVLVVSSADAAREIIKTNDLVFSDRPSLSVFYKLFYNGR

CYP71AV1 A. annua DVVLAPYGEYWRQLRKICTLELLSVKKVKSFQSLREEECWNLVQEIKASGSGRPVNLSEN

CYP71A1 S. melongena DVGFSPFGEYWWQVRSIVVLHLLSNKRVQSYRDVREEETANMIEKIRQGCDASVINLGEH

CYP71AJ14 T.garganica DVAFAPYTEYWRQVKSICVLQLLSNKRVRSFYNVREEEVGLLVENIKDSGS-NIVNLSDL

CYP71AJ12 T.laciniata DVAFAPYTEYWRQVKSICVLQLLSNKRVRSFYNVREEEVGLLVENIKDSGS-NIVNLSDL

CYP71AJ13 P.sativa DVAFAPYTEYWRQVKSICILQLLSNKRVRSFHNVREEEVGLLVENIRNSGS-EIVNLSDL

CYP71AJ6 T.laciniata DVVFAPYSEYWRQVKSICVLQLLSNKRVRSFQTVREEEVALLVENIRESGS-KTVNLSEL

CYP71AJ5 T.garganica DVVFAPYSEYWRQVKSICVLQLLSNKRVRSFQTVREEEVALLVENVRESGS-KTVNLSEL

CYP71AJ15 L.siler DVVFAPYSEYWRQVKSICVLQLLSNKRVRSFQTVREEEVALLVENIRESGS-RTVNLSEL

CYP71AJ7 D.carota DVVFAPYSEYWRQVKSICVLQLLSNKRVRSFQTVREEEVALLVENIRESGS-KAVNLSEL

CYP71AJ9 P.crispum DVVFAPYSEYWRQVKSICVLQLLSNKRVRSFQAVREEEVALLVENIKESGS-KPVNLSEL

CYP71AJ11 H.mantegazzium DVVFAPYSEYWRQVKSICVLQLLSNKRVRSFQIVREEEVALLVEKIKESGS-KPVNLSEL

CYP71AJ8 A.majus DVVFAPYSEYWRQVKSICVLQLLSNKRVRSFQTVREEEVALLIENIKESGS-KPVNLSEL

CYP71AJ3 P.sativa DMVFARYTEYWRQVKSTCVTQLLSVKRVQSFHNVREEEVALLLDNIENSKS-KVINLSEM

CYP71AJ27 H.lanatum DMVFARYTEYWRQVKSTCVTQLLSVKRVQSFHNVREEEVARLLQNIENSKS-KVMNLSEM

CYP71AJ1 A.majus DMVFARYTEYWRQVKSICVTQLLSNKRVNSFHYVREEEVDLLVQNLENSHS-KVANLTEL

CYP71AJ2 A.graveolens DMVFARYTEYWRQVKSICVTQLLSNKRVNSFQNVREEEVDLLVQNIENSCS-KVINLTEL

CYP71AJ4 P.sativa DVAFSRYSEYWRQVRSTCVTQLLSNSRVHSFHNIREEEVALLIQNIENSAS-EVINLGEQ

CYP71AJ21 D.carota DVAFARYSEYWRQVKSMCVTQLLSSRRVQSFHNVREEEVALLIQNIEHPPS-KIVNLSDL

CYP71AJ25 T.garganica DVAFALYSEYWRQVKSMCVTQLLSSRRVHSFHNVREEEVALLIRNIEYPPS-KIVNLSDL

CYP71AJ40 A.graveolens DVAFARYSEYWRQVKSTCITQLLSSSRVHSFHNVREEEVALFIQNIEDSYS-IIINLSDL

CYP71AJ32 B.chinensis DVGFSRYSEYWRQVKSICVAQLLSSKRVHSFQSVREEEVDLLIQNIQYSDL-NTINLSDM

CYP71AV1 A. annua IFKLIATILSRAAFGKGIKDQK------ELTEIVKEILRQTGG-FDVADIFPSKKFLHHL

CYP71A1 S. melongena LCFLTNNITSRVALGRTYDERESGI---DAKDILEQFLQLLDT-FNVGDYIPWLKWVNKI

CYP71AJ14 T.garganica TYTLLSNVVSRIALGKRYTNSTEDGEENSFRELFQNIAQLIGY-FSFSDYIPWLYWIDSL

CYP71AJ12 T.laciniata TYTLLSNVVSRIALGKKYTNSTEDGEENSFRELFQNIAQLIGY-FSFSDYIPWLYWIDSL

CYP71AJ13 P.sativa FYTLLSNVVSRIALGKKYTNTTEGGEENSFRELFQNIAQLIGY-FSFSDYIPWLYWIDSL

CYP71AJ6 T.laciniata FYTLLSNVVSRIALGRKYAITTEGGKENSFKELFQNIAQLIGY-VSVGDYIPWLFWIDSV

CYP71AJ5 T.garganica FYTLLSNVVSRIALGRKYAITTEGGKENSFKELFQNIAQLIGY-FSVGDYIPWLFWIDSV

CYP71AJ15 L.siler FYTLLSNVVSRIALGRKYAITTEGGKENAFKEVFQNIAQLIGY-FSVGDYIPWLFWVDSV

CYP71AJ7 D.carota FYALLSNVVSRIALGRKYAITTEGGKGNSFKELFQNIAQLIGY-FSVGDYIPWLFWIDSV

CYP71AJ9 P.crispum FYALLSNVVSRIALGRKYSIISEGGKESSLKELFQSIAQLIGY-FSVGDYIPWLFWIDSL

CYP71AJ11 H.mantegazzium FYALLSNVVSRIALGRKYGITTEGGKDNSFKELFQNIAQLIGY-FSVGDYIPWLFWVDSL

CYP71AJ8 A.majus FYALLSNVVSRIALGRKYAIITEGGKESSFKELFQSIAQLIGY-LSVGDYIPWLFWVDSL

CYP71AJ3 P.sativa LIELTGNVVCRAALGSGYNVD-------SYKSLLLQIMDMLGYSRSIEDFFPSLGWVDWI

CYP71AJ27 H.lanatum LIELTGNVVCRAALGSGYNVD-------SYKSLLLQIMDMLGYSRSIEDFFPSLGWVDWI

CYP71AJ1 A.majus LIEVTGNVVCRVSVGSGDKVD-------SYKILILEIMDMLGYSRSIEDFFPLLGWVDWL

CYP71AJ2 A.graveolens LIEVTGNVVCKVSVGSGDKVD-------SYKILILEIMEMLGYSRSIEDFFPMFGWVDWL

CYP71AJ4 P.sativa LIQLTRNVVCRVSVGSEYLSGHKGK---LYQKLLAEVTEMLAYTYSIGDFIPLLGWVDWL

CYP71AJ21 D.carota LAELAQNVVCRVALGRKYGRGIDGNS--SYKILLGEIMELIGYSRSMRDFFPLLGWVDRL

CYP71AJ25 T.garganica LAELTQNVVCRVALGRKYESGDKGN---SYKILLGEIMELLGYSRSMGDFFPLLGWVDWL

CYP71AJ40 A.graveolens LAELTKNVVCRVALGRKYENGHKGN---SFKSLLGDIMNMLGYSRSIGDFFPFFGWVDWL

CYP71AJ32 B.chinensis FSELANNVVCRVSLGRKYSDKGN-----SYNRSLGKVMELLGS-RSIGDYIPLLCWVDLL

CYP71AV1 A. annua SGKRARLTSLRKKIDNLIDNLVAEHTVNTSSKTNE------TLLDVLLRL---KDSAEFP

CYP71A1 S. melongena TGLDTKVEKIAKKLDTFLDSVIEEHIIRNKKEEYAITDEAKDFVDVLLEIQ-NGKETDFP

CYP71AJ14 T.garganica NGLKGRVEKAANEIDAFLESVIRDHSTAL---TNGASSD--DLLKTLLEIEKQDSNSAFS

CYP71AJ12 T.laciniata NGLKGRVEKAANEIDAFLEGVIRDHSTAL---INGASSD--DLLKTLLEIQKQDSNSAFS

CYP71AJ13 P.sativa SGLKKRVEKAANEIDAFLEGVIRDHSIAL---STGASSD--DLLNTLLEIQKQDTNSAFS

CYP71AJ6 T.laciniata NGLKGRVEKAANEADLFLEGVIKDHSVAL---DNGASSD--DLLYNLLEIQKQDTNSTFS

CYP71AJ5 T.garganica NGLKGRVEKAANEADLFLEGVIKDHSIAL---DNGVSTD--DLLYNLLEIQKQDTNSAFS

CYP71AJ15 L.siler TGLKGRVEKAANEVDLFLEGVIKDHSIAL---DNGASSD--DLLYNLLEIQKQDTNSAFS

CYP71AJ7 D.carota SGLKGRVEKAANEADLFLESVIKDHSIAL---DNGASSD--DLLYNLLEIQKQNTNSAFS

CYP71AJ9 P.crispum NGLKGQVEKASAEVDVFLEGVIRDHRIAL---DNGASSD--DLLYNLPEIQKQNTNSAFS

CYP71AJ11 H.mantegazzium NGLKGRVEKASTEVDVFLEGVIRDHSVAL---DNGASSD--DLLYNLLEIQKQNTNSAFS

CYP71AJ8 A.majus SGLKGRVEKASAEVDVFLEGVIRDHLIAL---ENGASRD--DLLYNLLEIQKQNNGSAFS

CYP71AJ3 P.sativa TGLKGKVEKAANGVDAFLEGVLKNHTNPSTSSANK------DFVSILLEI--QEADAGSS

CYP71AJ27 H.lanatum TGLKGKVEKAANGVDAFLEGVLKNHTNPSTSASNNSANK--DFVSILLEI--QEADAGSS

CYP71AJ1 A.majus TGLRGKVAEAAKGVDTFLEGVLKEHLSTTGSKYN-------DFVSILLEI--QEADAGSS

CYP71AJ2 A.graveolens TGLRGKVAKAAKGVDDFLEGVLKEHLTARAS-NNASADN--DFVSILLEI--QEADAGST

CYP71AJ4 P.sativa SGSKAKVEKTAKEVDAFLEGALRDHIKTMAS-NKGSAND--DFLSILLEI--READAGST

CYP71AJ21 D.carota TGLNARAEKAAKEVDTFLEGVLRDHPSTVAS-NNGHANK--DFVSILLEI--QNTDAGSS

CYP71AJ25 T.garganica NGLKAKVEKAANEVDTFLEGVLRDHPSTVAS-NNGYANK--DFVSILLEI--QNTDAGSS

CYP71AJ40 A.graveolens NGLNGKVEKLAKEMDTFLEGVLRDRLSTSAL-NIDSANK--DFVSILIDI--QNTDAASS

CYP71AJ32 B.chinensis TGLKGKVEKVAKEVDAFLEGVLREHESTVASGTDGCGNG--NFISILLDVQKQNTDNGFS

CYP71AV1 A. annua LTSDNIKAIILDMFGAGTDTSSSTIEWAISELIKCPKAMEKVQAELRKALNGKEKIHEED

CYP71A1 S. melongena LQRDSLKAILLDAFAAGTDTIYTNLDWTMADVLRQPRAMKTLQNEVRGLAQGKSEITEDD

CYP71AJ14 T.garganica IDKDSIKGVILNMYFDGTDSTSAVLEWTMAALIKHPDIMCKLKNEVREIGRGKSRINGDD

CYP71AJ12 T.laciniata IDKDSIKGVILNMYFDGTDSTSAVLEWTMAALIKHPDIMCKLKNEVREIGRGKPRINGDD

CYP71AJ13 P.sativa IDNDSIKGVILNMYFDGTDSTSAVLEWTMAALIKHPDIMCKLKDEVREIGRGKSRISGDD

CYP71AJ6 T.laciniata IDKDSIKGVILNMFFDGTDTTSAVLEWTMAALIKHPDIMCKLKNEVREIGRGKSKICGDD

CYP71AJ5 T.garganica IDKDSIKGVILNMFFDGTDTTSAVLEWTMAALIKHPDIMCKLKNEVREIGRGKSKISGDD

CYP71AJ15 L.siler IDKDSIKGVILNMFFDGTDTTSAVLEWTMAALIKHPDIMCKLKNEVREIGRGKSKISGDD

CYP71AJ7 D.carota IDKDSIKGVILNMFFDGTDTTSAVLEWTMAALIKHPDVMCKLKNEVREIGRGKLRINGDD

CYP71AJ9 P.crispum IDKDSIKGVILNMFFDGTDTTSAVLEWTMAALIKNPDIMRKLQNEVREIGRGKSTISGDD

CYP71AJ11 H.mantegazzium IDKDSIKGVILNMFFDGTDTTSAVLEWTMAALIKHPDIMRKLQNEVRDIGRGKSTISGDD

CYP71AJ8 A.majus IDKDSIKGVILNMFFDGTDTTSAVLEWTMAALIKNPDIMHKLQSEVREIGRGKSTISGDD

CYP71AJ3 P.sativa MDKECIKSLIWDMLGAGTETIATALEWTIGALIKSPDAMSKLQKEVREIGKGKSRIEEGD

CYP71AJ27 H.lanatum MDKECIKSLIWDMLGAGTETIATALEWTIGALIKSPDAMSKLQKEVREIGKGKSRIEEGD

CYP71AJ1 A.majus MDNECIKSLIWDMLGAGTETISTALEWTLAALIKNPDAMFKLQNEVREIGKGKSKISEAD

CYP71AJ2 A.graveolens MDNECIKSLIWDMLGAGTETISTALEWTLAALIKNPDAMLKLQNEVREIGKGKSKISEAD

CYP71AJ4 P.sativa LDEECIKAIVWDMILGGTETTSTTLEWIVAAIIKNPDVMFKLQKEVREIGKGKSKIEEVD

CYP71AJ21 D.carota MDKDCIKAVIWDMFVAGTDTTSSTLEWAIAALIKNPHVMVKLQNEVREIGKGKSKISEDD

CYP71AJ25 T.garganica MDKDCIKALIWDMFGAGTDTTSTTLEWTIAALIKSPDVMVKLQKEVREIGRGKSNISEDD

CYP71AJ40 A.graveolens IDKECIKAVILDMFVAGTETTATSLEWTIAALIKSPDVMFKLQNEVREIGNGKSMILEGD

CYP71AJ32 B.chinensis IDKDCIKAVILDMIVAGTDTTSTTLEWTIAALIKSPDAMFKLQKEVREIGKGKSKISEDD

CYP71AV1 A. annua IQELSYLNMVIKETLRLHPPLPLVLPRECRQPVNLAG-YNIPNKTKLIVNVFAINRDPEY

CYP71A1 S. melongena LKNMQYLRAVIKESLRLHPPNSLLVPRESMEDVKLLGYYHIPARTQALINVWAIGRDPLS

CYP71AJ14 T.garganica LEQMHYLRAVIKESMRLYTPVPLLVAREAMQDVKVMG-YDIKAGTQVLINAWAIATDPAV

CYP71AJ12 T.laciniata LEQMHYLRAVIKESMRLYTPVPLLVAREAMQDVKVMG-YDIKAGTQVLINAWAIATDPAV

CYP71AJ13 P.sativa LEKMHYLRAVIKESMRIYTPVPLLVAREAMQDVKVMG-YDIKAGTQVLINAWAIATDGAV

CYP71AJ6 T.laciniata LEKMHYLKAVVKESMRIYTPVPLLVAREAMQDVKLMG-YDVKSGTQVLINAWAIATDPAL

CYP71AJ5 T.garganica LEKMHYLKAVVKESMRVYTPVPLLVAREAMQDVKVMG-YDVKAGTQVLINAWAIATDPAL

CYP71AJ15 L.siler LEKMHYLKAVVKESMRIYTPVPLLVAREAMQDVKVMG-YDVKAGTQVLINAWAIATDPAL

CYP71AJ7 D.carota LENMHYLKAVIKESMRLYTPVPLLVAREAMQDVKVMG-YDVKDGTQVLINAWAIATDPAL

CYP71AJ9 P.crispum LENMHYLKAVIKESMRIYTPVPLLVAREAMQDVNVMG-YYIKAGTQVMINAWAIATDPTL

CYP71AJ11 H.mantegazzium LENMHYLKAVIKESMRIYTPVPLLVAREAMQDVNVMG-YDIKAGTQVLINAWAIATDPTL

CYP71AJ8 A.majus LENMHYLKAVIKESMRICTPVPLLVAREAMQDVNVMG-YDIRAGTQVMINAWAIATDPTV

CYP71AJ3 P.sativa LVKMDYLKAVMKESMRLYFTAPLLVPREARQDVKFMG-YDIKSGTQVLINAWAIARDPSS

CYP71AJ27 H.lanatum LVKMDYLKAVMKESMRLYFTAPLLVPREARQDVKFMG-YDIKSGTQVLINAWAIARDPAS

CYP71AJ1 A.majus LVKMNYLQAVMKESMRLYFTAPLLVPREARQDIKFMG-YDISSGTQVLINAWAIARDPLL

CYP71AJ2 A.graveolens LGKMTYLQAVMKESMRLYFTAPLLVPRESRQDVKFMG-YDISAGTQVLINVWAIARDPSL

CYP71AJ4 P.sativa LVKMNYLKAVMKESMRLYITA-FLLPREAKQDVKLMG-YDISSGTQVLINTWETARDPSL

CYP71AJ21 D.carota IVKMNYLKAVMKESMRLYMTAPLIVPREARQDVKVMG-YDIRKGTQLVINAWAIARDPSL

CYP71AJ25 T.garganica LVKMNYLKAVIKESMRLYFTAPLIVPREARQDVKVMG-YDIRKGTQVLINAWAIARDPSL

CYP71AJ40 A.graveolens LVKMNYLKAVMKESMRLYFTAPLLVPRQARQDVKVMG-YDIRSGTQVLINAWAIARDPSL

CYP71AJ32 B.chinensis LKNMHYLKAVMKESMRLNFTAPFLLPREARKDVKVMG-YDIRSGTQVIINAWAIARDPSL

CYP71AV1 A. annua WKDAEAFIPERFENSSATVMGAEYEYLPFGAGRRMCPGAALGLANVQLPLANILYHFNWK

CYP71A1 S. melongena WENPEEFCPERFLNNDIDMKGLKFELLPFGSGRRGCPGSSFAIAVIELALARLVHKFNFA

CYP71AJ14 T.garganica WDNPGEFIPERFLNNSVDYKGLHFEFIPFGAGRRGCPGIQYAMAINELALATLVHIFDFA

CYP71AJ12 T.laciniata WDNPEEFIPERFLNNSVDYKGVHFEFIPFGAGRRGCPGIQYAMAINELALANLVHIFDFA

CYP71AJ13 P.sativa WDNPEEFIPERFLNNPIDYKGLHFEFIPFGAGRRGCPGIQYAMAINELALANLVHIFDLA

CYP71AJ6 T.laciniata WDNPEEFIPERFLNNPIDYKGLHFEFIPFGAGRRGCPGIQYAMAINELALANLVHIFDFA

CYP71AJ5 T.garganica WDNPEKFIPERFLNNPIDYKGLHFEFIPFGAGRRGCPGIQYAMAINELALANLVHIFDFA

CYP71AJ15 L.siler WDNPEEFIPERFLNNPTDYKGLHFEFIPFGAGRRGCPGIQYAMAINELALANLVHIFDFA

CYP71AJ7 D.carota WDNPEEFIPERFLNNPIDYKGLHFEFIPFGAGRRGCPGIQYAMAINELAVANLVHIFDFA

CYP71AJ9 P.crispum WDNPEEFIPERFLNNPVDHKGMHFEFIPFGARRRGCPGIQYAMAINELALANLVHIFDFA

CYP71AJ11 H.mantegazzium WDNPEEFIPERFLNNPVDYKGLHFEFIPFGAGRRGCPGIQYAMAINELALANLVHIFDFA

CYP71AJ8 A.majus WDKPEEFIPERFLNNPLDYKGLHFEFIPFGAGRRGCPGIQYAMAINELALANLVHIFDFA

CYP71AJ3 P.sativa WDNPEEFRPERFLNSPIDYKGFNYEYIPFGAGRRGCPGIQFAISVNELVVANVVNKFNFE

CYP71AJ27 H.lanatum WDNPEEFLPERFLNSPIDYKGFNYEYIPFGAGRRGCPGIQFAISVNELVVANVVNKFNFE

CYP71AJ1 A.majus WDKPEEFRPERFLNSPIDYKGFHYEFLPFGAGRRGCPGIQFAMCINELVVANLVHKFNFE

CYP71AJ2 A.graveolens WEKPEEFRPERFLNSHIDYKGFNYEYLPFGAGRRGCPGIQFAMAVNELVVANVIHKFNFE

CYP71AJ4 P.sativa WDNPEEFRPERFLNSPIDYKGLHYEYLPFGGGRRGCPGIQFAMAVNELAVANVVYKFDFK

CYP71AJ21 D.carota WDSPEEFRPERFLNSPIDYKGLHYEYLPFGAGRRGCPGYHFAMAVNELALANVVHKFDFE

CYP71AJ25 T.garganica WENPEKFQPERFLNSPIDYKGLHYEYLPFGGGRRGCPGIQFAMAVNELAVANVVHKFDFE

CYP71AJ40 A.graveolens WDKPEEFRPERFLNSPIDYKGLHYEYVPFGAGRRGCPGIQFAMAVNELAVANVVHKFDFK

CYP71AJ32 B.chinensis WNNPEEFRPERFLDSPIDYKGVHYEFIPFGGGRRMCPGIQFAMAVNELAIANIVHKFDFE

CYP71AV1 A. annua LPNGVSYDQIDMTESSGATMQRKTELLLVPSF---

CYP71A1 S. melongena LPKGTKPEDLDMTECTGIATRRKSPLPVVATPFSG

CYP71AJ14 T.garganica LPDGKRFEDLDMASETGMTVHKKSPLLVIATPRI-

CYP71AJ12 T.laciniata LPDGKRFEDLDMASETGMTVHKKSPLLVIATPRI-

CYP71AJ13 P.sativa LPDGKRFENLDMDSETGMTVHKKSPLLVIATPCI-

CYP71AJ6 T.laciniata LPDGRRLEDLDMTSETGMTLHKKSPLLVIATSRV-

CYP71AJ5 T.garganica LPDGRRLEDLDMTSETGMTLHKKSPLLVIATSRV-

CYP71AJ15 L.siler LPDGRRLEDLDMTSETGMTLHKKSPLLVIATSRV-

CYP71AJ7 D.carota LPDGRRLEDLDLTSETGMTLHKKSPLMVIATSRV-

CYP71AJ9 P.crispum LPGGKRLEDLDMDAETGMTLHKKSPLLVVATSRV-

CYP71AJ11 H.mantegazzium LPDGKRFEDLDMTAETGMTLHKKSPLLAIATSRV-

CYP71AJ8 A.majus LPDGKRFEDLDMDAETGMTLHKKSPLLVIATSRV-

CYP71AJ3 P.sativa LPDGKRLEEMDMTASTGITFHKKSPFFVVATPHV-

CYP71AJ27 H.lanatum LPDGKRLEEMDMTASTGITFHKKSPIFVVATPHV-

CYP71AJ1 A.majus LPDGKRLEDLDMTAASGITLRKKSPLLVVARPHV-

CYP71AJ2 A.graveolens LPDGERLEDLDMTAVSGITLRKKSPLLVVARPHV-

CYP71AJ4 P.sativa MPDGERFEDLDMSGVPGISLYRKYPLMVVASPHV-

CYP71AJ21 D.carota LPNGERKEDLDMTGVTGLTVRRKSPLLVIATPHV-

CYP71AJ25 T.garganica LPNGETREDLDMTGVTGITLRRKSPLLVIATPHV-

CYP71AJ40 A.graveolens LPDGKRLEDLDMTAVTGLTLRRESPLLVVATPHI-

CYP71AJ32 B.chinensis LPDGERLQDLDMTCVTGVTLCRKSPLLVIAKPHV-
